# Repetitive head impacts induce neuronal loss and neuroinflammation in young athletes

**DOI:** 10.1101/2024.03.26.586815

**Published:** 2024-03-28

**Authors:** Morgane L.M.D. Butler, Nida Pervaiz, Petra Ypsilantis, Yichen Wang, Julia Cammasola Breda, Sarah Mazzilli, Raymond Nicks, Elizabeth Spurlock, Marco M. Hefti, Bertrand R. Huber, Victor E. Alvarez, Thor D. Stein, Joshua D. Campbell, Ann C. McKee, Jonathan D. Cherry

**Affiliations:** 1 Department of Anatomy & Neurobiology, Boston University Chobanian & Avedisian School of Medicine, Boston MA, USA; 2 Boston University Alzheimer’s Disease and CTE Centers, Boston University Chobanian & Avedisian School of Medicine, Boston MA; 3 Section of Computational Biomedicine, Department of Medicine, Boston University Chobanian & Avedisian School of Medicine, Boston MA, USA; 4 VA Boston Healthcare System, Jamaica Plain MA, USA; 5 VA Bedford Healthcare System, Bedford MA, USA; 6 Department of Pathology, University of Iowa Health Care, Iowa City IA, USA; 7 National Center for PTSD, VA Boston Healthcare System, Boston MA, USA; 8 Department of Neurology, Boston University Chobanian & Avedisian School of Medicine, Boston MA, USA; 9 Department of Pathology and Laboratory Medicine, Boston University Chobanian & Avedisian School of Medicine, Boston MA, USA

## Abstract

Repetitive head impacts (RHI) sustained from contact sports are the largest risk factor for chronic traumatic encephalopathy (CTE). Currently, CTE can only be diagnosed after death and the multicellular cascade of events that trigger initial hyperphosphorylated tau (p-tau) deposition remain unclear. Further, the symptoms endorsed by young individuals with early disease are not fully explained by the extent of p-tau deposition, severely hampering development of therapeutic interventions. Here, we show that RHI exposure associates with a multicellular response in young individuals (<51 years old) prior to the onset of CTE p-tau pathology that correlates with number of years of RHI exposure. Leveraging single nucleus RNA sequencing of tissue from 8 control, 9 RHI-exposed, and 11 low stage CTE individuals, we identify SPP1+ inflammatory microglia, angiogenic and inflamed endothelial cell profiles, reactive astrocytes, and altered synaptic gene expression in excitatory and inhibitory neurons in all individuals with exposure to RHI. Surprisingly, we also observe a significant loss of cortical sulcus layer 2/3 neurons in contact sport athletes compared to controls independent of p-tau pathology. These results provide robust evidence that multiple years of RHI exposure is sufficient to induce lasting cellular alterations that may underlie p-tau deposition and help explain the early clinical symptoms observed in young former contact sport athletes. Furthermore, these data identify specific cellular responses to repetitive head impacts that may direct future identification of diagnostic and therapeutic strategies for CTE.

## Introduction

Each year, millions of individuals are exposed to repetitive head impacts (RHI) through contact sports, military service, and domestic violence. These RHIs are often non-symptomatic, non-concussive, and can occur thousands of times per year, over the course of decades in some cases. Chronic traumatic encephalopathy (CTE), a progressive tauopathy caused by exposure to RHI, is observed in individuals as young as 17^1,2^. Risk for CTE in exposed individuals is associated with the number of years of exposure to RHI and the cumulative force of the hits endured^3,4^. While much of the current research is focused on severe CTE in older individuals, a recent case series of 152 brains from donors under the age of 30 identified 63 brains with CTE, highlighting that RHI-driven disease is also pressing concern in the young population^2^. Currently, CTE can only be diagnosed postmortem through identification of hyperphosphorylated tau (p-tau) aggregates in neurons around blood vessels at the depth of the cortical sulcus. Our previous research suggests that microglia-mediated neuroinflammation occurs prior to the deposition of p-tau^5^. Additionally, other work has demonstrated RHI exposure is associated with astrocytic activation, white matter inflammation and damage, blood-brain barrier (BBB) breakdown, serum protein leakage, and increases in vascular density in the CTE brain^5–9^. These cellular changes occur prior to overt neurodegeneration and are likely driving many of the early clinical impairments not explained by the occurrence and extent of p-tau pathology. However, studies examining the full extent of these cellular phenotypes have been limited. A detailed characterization of the early cellular changes in young RHI-exposed athletes is necessary to understand the pathogenic mechanisms in CTE and to identify novel biomarkers or therapeutic targets relevant to early disease stages.

## Results

### Cell type identification and cell proportion analysis across pathological groups

**Extended Data Figure 1.**
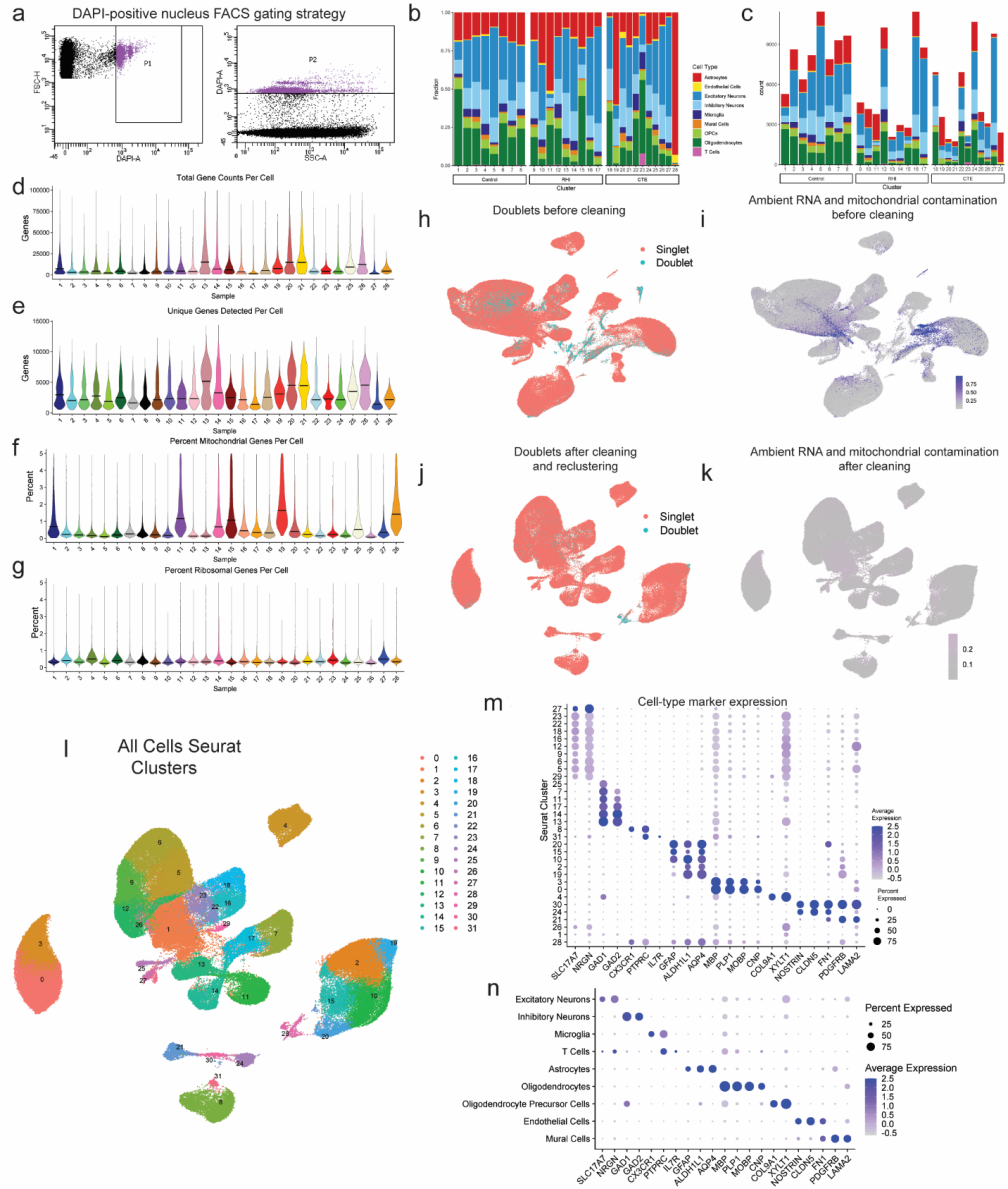
Dataset quality control and cell type marker validation. **a.** Fluorescence activated cell sorting gating strategy of DAPI-positive nuclei. **b.** Stacked bar plot representing the proportion of cell type per donor. **c.** Stacked bar plot representing the cell type counts per donor. **d-e.** Violin plots for each donor of **(d)** total gene counts per cell, **(e)** unique genes detected per cell, **(f)** percent of mitochondrial genes detected per cell, and **(g)** percent ribosomal genes detected per cell. Line represents median. **h,i** UMAP of full dataset before cleaning colored by **(h)** doublet or singlets or **(i)** mitochondrial contamination. **j,k.** UMAP of full dataset after cleaning colored by **(j)** doublets or singlets or **(k)** mitochondrial contamination. **l.** UMAP of full dataset colored by Seurat clusters. **m.** Dot plot of cell type marker expression across Seurat clusters depicted in **(l)**. **n.** Dot plot of cell type marker expression in annotated cell type clusters.

**Figure 1.**
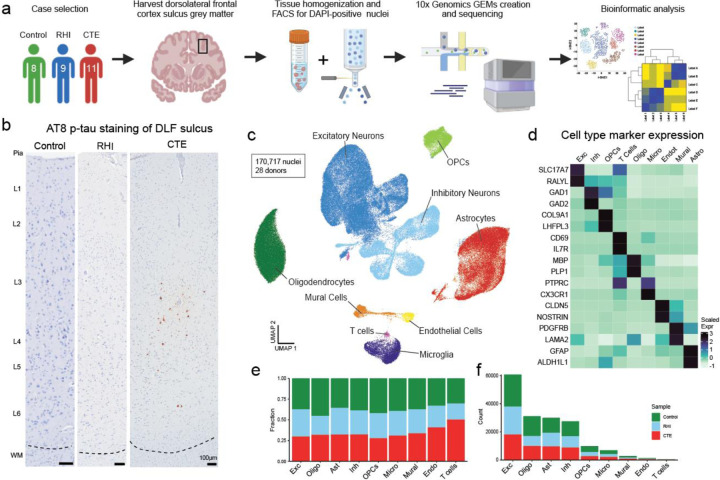
Cell type identification and cell proportion analysis across pathological groups. **a.** Diagram depicting experimental workflow. **b.** AT8 immunohistochemistry of dorsolateral frontal cortex depth of sulci, dashed line represents the grey-white matter interface. Scale bar, 100μm. **c.** UMAP of nuclei from all donors labelled for cell type based on cell-type marker expression. **d.** Expression of cell type markers across cell type clusters in (c). **e.** Stacked bar plot of pathological group fractions within cell type clusters. **f.** Stacked bar plot of cell type counts colored by pathological group.

To identify the earliest RHI driven changes, we performed single nucleus RNA sequencing (snRNAseq) using autopsy-confirmed frozen human brain tissue from 28 young individuals. 8 non RHI-exposed controls, 9 RHI-exposed individuals without CTE pathology, and 11 RHI-exposed individuals with diagnosed CTE stage 1 or 2 were included (Fig. 1a, Supplementary Table 1,2). CTE diagnosis was performed by a neuropathologist and based on the presence of CTE pathognomonic p-tau lesions^10^ (Fig. 1b). Grey matter sulcus from the dorsolateral frontal cortex, one of the first brain regions affected in CTE, was processed for snRNAseq (Fig. 1a). After filtering, 170,717 nuclei of sufficient quality were clustered into 31 initial clusters and labeled based on expression of known cell type markers^11,12^ (Fig 1c, Extended Data Fig 1l–n). All major cell types were identified. Compositional analysis demonstrated no significant differences in cell type abundance across pathological groups^13^ (Fig 1d–f, Supplementary Fig. 1a). Significant RHI-associated cellular phenotypes were observed in microglia, astrocytes, endothelial cells, and neurons, therefore analysis was performed on these cell types. Few RHI or CTE-specific subtypes and differentially expressed genes (DEG) were identified in oligodendrocytes, oligodendrocyte precursor cells, or T cells (Supplementary Fig. 1b–k), likely indicating that in this dataset RHI-induced cellular changes are less prominent in these cell types in the grey matter of young individuals.

### RHI exposure induces distinct microglial phenotypes

**Figure 2.**
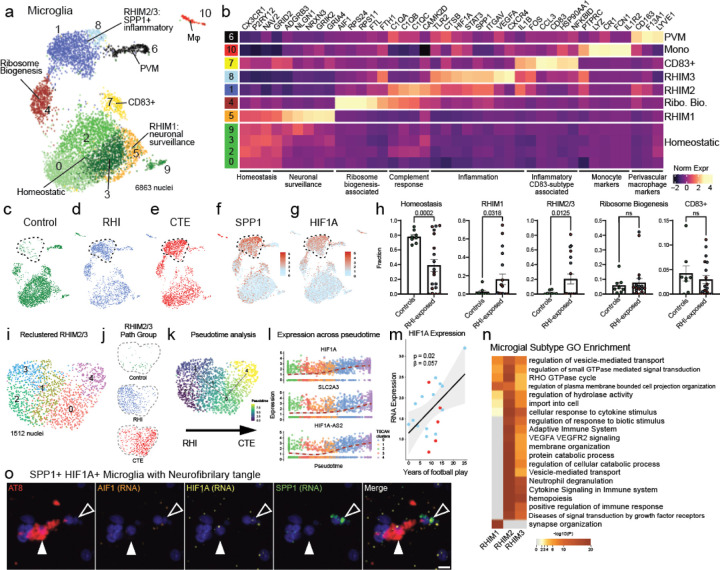
RHI Exposure induces distinct microglial phenotypes. **a.** UMAP of microglia colored by 10 Seurat clusters determined by unsupervised clustering. **b.** Heatmap of selected cluster DEGs annotated by function. **c-e,** UMAP in (a) subsetted for pathological group identity, dashed lines encircling RHIM2/3. **f,g.** UMAP in (a) colored by SPP1 and HIF1A expression, dashed lines encircling RHIM2/3. **h.** Bar plots showing cluster distribution in control and RHI-exposed samples, dots represent individual donors and are colored by pathological group identity. Bars represent mean, error bars standard error of the mean, statistical analysis was performed with a two-tailed t-test with Welch’s correction. **i.** UMAP showing reclustered RHIM2/3 colored by 4 Seurat clusters. **j.** UMAP in (i) colored by pathological group. **k.** UMAP in (i) colored for pseudotime analysis and demonstrating the shift in RHI/CTE propensity across pseudotime. **l.** Expression of selected DEGs significantly associated with pseudotime trajectory, statistics performed with singleCellTK package, FDR<0.05, log2Fc >0.15. **m.** Scatter plot depicting HIF1A expression in SPP1+ HIF1A+ cells from *in situ* hybridization compared to years of football play. Statistics performed using simple linear regression. **n.** Heatmap depicting GO terms identified in upregulated genes in RHIM1-3. **o.** Representative image of *in situ* hybridization with protein codetection of AT8 protein (red), AIF RNA (orange), HIF1A RNA (yellow), SPP1 RNA (green), solid arrows indicate neurofibrillary tangle, open arrows indicate SPP1/HIF1A/AIF1+ cell. Scale bar 10µm.

Based on previously demonstrated involvement of microglial inflammation in CTE and its important role in neurodegeneration, we first examined microglial gene expression changes^5^. Analysis of microglial cells revealed eleven unique clusters (Fig. 2a). Cluster 10 expressed perivascular macrophage (PVM) genes CD163, F13A1, and LYVE1 and cluster 6 expressed peripheral monocyte genes PTPRC, LYZ, and CR1^11,14,15^ (Fig. 2b). Clusters 0, 2, 3, and 9 expressed classical microglial homeostatic genes CX3CR1, P2RY12, and NAV2 and were labelled as homeostatic microglia. Homeostatic clusters were significantly enriched for nuclei from control individuals (p=0.0002, Fig. 2h). Cluster 7 highly expressed CD83, CCL3, and HSP90AA1, reminiscent of a possible pro-resolving phenotype recently identified in Alzheimer’s disease (AD)^16^. Cluster 4 expressed the greatest AIF1 (Iba1) across clusters and was characterized by expression of FTL and FTH1 iron-associated genes along with expression of ribosomal-associated genes such as RPS24 and RPS11 (Fig. 1b).

**Extended Data Figure 2.**
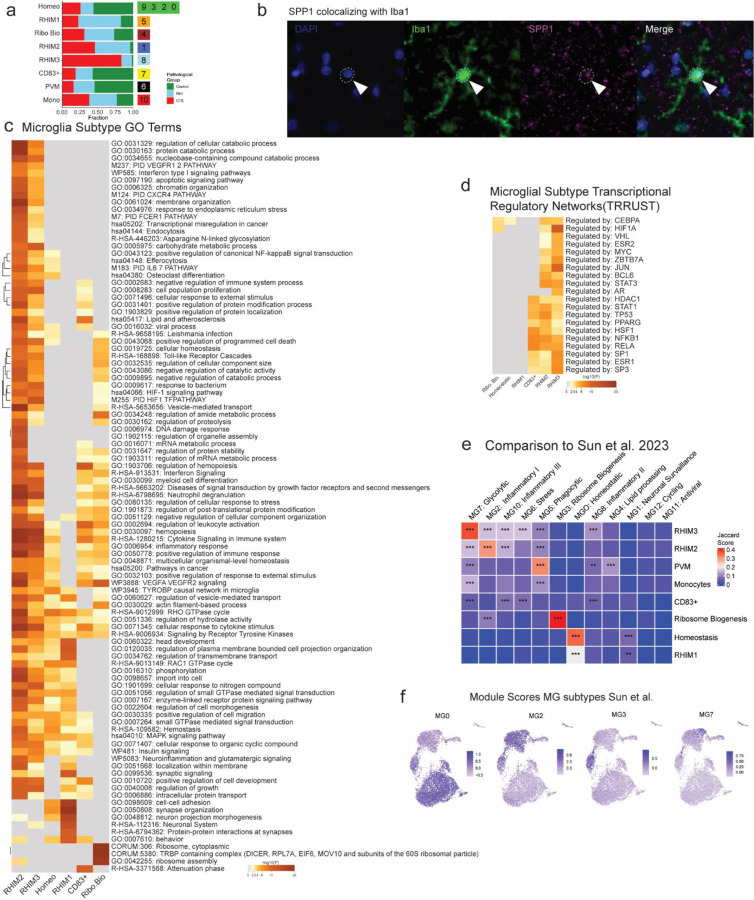
Microglial cluster GO analysis, histology, and validation. **a.** Stacked bar plot of microglial subtype abundance across pathological groups. **b.** Fluorescent immunohistochemical staining for Iba1 (green), SPP1 (pink), dashed line depicting microglial soma, solid arrow indicating colocalized SPP1 and Iba1. Scale bar, μm. **c.** Heatmap of top 100 GO terms for microglial subtype DEGs. **d.** Heatmap of transcriptional regulatory network analysis of microglial subtype DEGs. **e.** Heatmap depicting Jaccard score similarity analysis between Sun et al. and current study microglial DEGs. **, p<0.01, ***, p<0.001. Statistical analysis performed using GeneOverlap package and Jaccard analysis settings. **f.** UMAPs depicting microglia colored for module scores of microglial subtypes from Sun et al.

Clusters 1, 5, and 8 were enriched for RHI-exposed samples (Fig. 2c–e,h, Extended Data Fig. 2a). For simplicity, these clusters were labeled repetitive head impact microglia (RHIM) 1 through 3. Cluster 5, RHIM1, expressed neuronal-associated genes such as GRID2, GRIK2, and GRIA4, with top identified gene ontology (GO) terms including “synapse organization” (Fig. 2b,n). Previous work has found that “satellite microglia” – microglia that closely contact neurons – increase in number following TBI and modulate neuronal firing activity^17^. The increase in neuronal surveillance microglia in RHI-exposed groups may represent a lasting increase in satellite microglia following RHI and in CTE, potentially contributing to synaptic dysfunction or altered neuronal firing.

Cluster 1, RHIM2, were nearly evenly enriched for RHI and CTE (50% vs 46%, respectively) while cluster 8, RHIM3, were mostly CTE-enriched (83%, Extended Data Fig 2a). Transcriptionally, RHIM2 and RHIM3 were similar, displaying features of an inflammatory microglial phenotype with expression of SPP1, HIF1A, TLR2, IL1B, and CTSB (Fig. 2b,f,g). SPP1 has been described as a general marker of inflammatory or activated microglia, potentially playing a role in synaptic engulfment in AD models^18^. SPP1 has also been described as an opsin for extracellular debris and our observed immunohistochemical staining was sparse and spotty, labelling Iba1+ microglia, but also appearing to speckle the extracellular space consistent with previous reports^19,20^ (Extended Data Fig. 2b). GO analysis of RHIM2/3 DEGs identified “cytokine signaling in the immune system”, “positive regulation of immune response”, and “vesicle mediated transport” (Fig. 2n, Extended Data Fig. 2c).

RHIM2 expressed C1QA, C1QB, C1QC, and CAMK2D the components and downstream effector of the C1q complement cascade known to drive aberrant synaptic engulfment in the neurodegenerative brain^21^. RHIM3 were characterized by upregulation of HIF1A and VEGFA, two central mediators of hypoxia, suggesting a potential response to or initiation of hypoxic conditions following RHI. HIF1A also acts as a transcriptional regulator of numerous downstream inflammatory genes, and analysis of the transcriptional regulatory networks enriched in each cluster showed that RHIM3 expressed many genes regulated by HIF1A^22^ (Extended Data Fig. 2d). To better understand the relationship between RHIM2 and RHIM3, reclustering and pseudotime analysis was performed on this subset. A transcriptional shift from RHIM2 to RHIM3 was correlated with a transition from RHI to CTE (Fig. 2i–k). HIF1A was significantly increased across the pseudotime trajectory along with HIF1A-AS2, the antisense gene for HIF1A and SLC2A3, a glucose transporter increased in microglia in response to hypoxia (Fig. 2l). These results suggest that RHIM2 and RHIM3 may represent cells along a continuum from RHI to CTE, as microglia become more inflammatory as disease processes progress. To verify the presence of RHIM2/3 cells and their relationship to pathology, *in situ* hybridization analysis was performed. SPP1/HIF1A+ cells showed increased expression of HIF1A with increasing number of years of football play (p = 0.02, β = 0.057, Fig. 2m). SPP1/HIF1A+ microglia associated with neurofibrillary tangles, suggesting a potential role in p-tau pathology (Fig. 2o). Finally, we sought to compare our microglial populations to those described in published datasets. Jaccard similarity scoring analysis demonstrated alignment of RHIM2/3 with inflammatory, stress, phagocytic, and glycolysis-associated populations ^22^ (Extended Data Fig. 2g,h). Overall, these results suggest that RHI exposure induces an increase in neuronal surveillance and inflammatory microglial transcriptomic states before the onset of CTE. These microglia may be involved in the initiation and maintenance of neuronal dysfunction, inflammation, and angiogenic processes present in CTE.

### Astrocytic responses to repetitive head impacts

**Figure 3.**
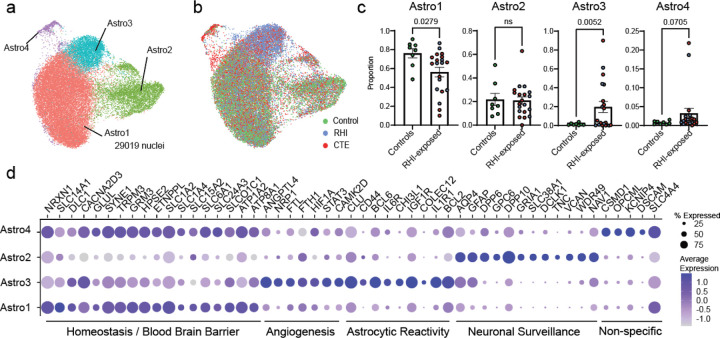
Astrocytic responses to head trauma. **a.** UMAP representing 4 astrocytic subtypes. **b.** UMAP from (a) colored by pathological group. **c.** Bar plots showing astrocyte subcluster distribution in control and RHI-exposed samples, dots represent individual donors colored by pathological group identity. Bars represent mean, error bars represent standard error of the mean. Statistical analysis was performed using two-tailed Mann Whitney U-test. **d.** Dot plot representing expression of selected DEGs across astrocytic subtype and annotated by function.

Astrocytes play a key role in brain homeostasis in tasks such as neuronal and BBB maintenance and become reactive following RHI exposure and in neurodegenerative disease ^23,24^. Four subtypes of astrocytes, Astro1-4, were identified based on stratification of pathological group identity and differentially expressed genes in each cluster (Fig. 3a).

**Extended Data Figure 3.**
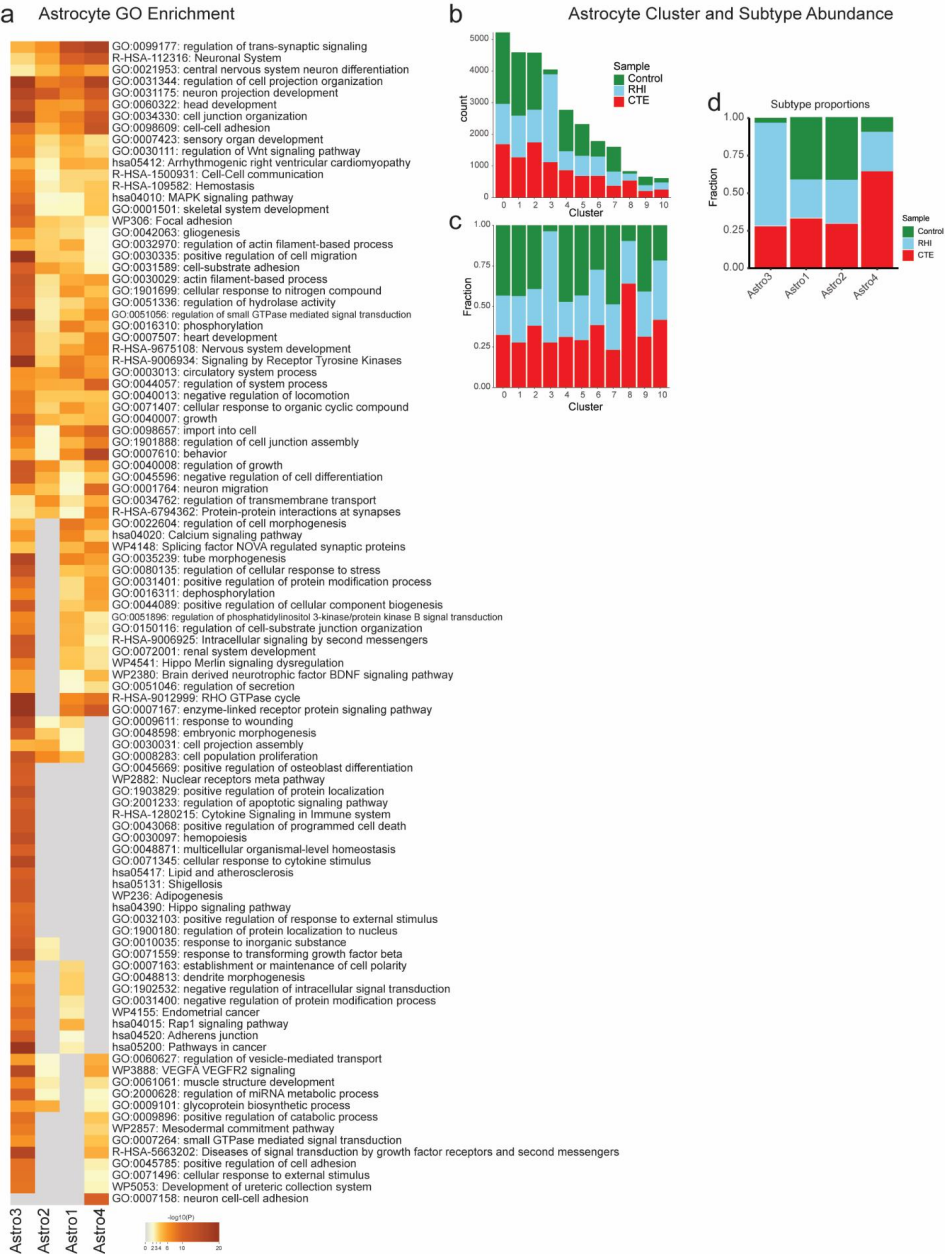
Astrocyte GO analysis and cell type proportions. **a.** Heatmap of top 100 go terms of astrocyte subtype DEGs. **b-d.** Stacked bar plots of astrocyte Seurat cluster (b) counts and (c) proportion by (b) pathological group, and astrocyte subtype proportions per pathological group.

Astro1 expressed genes involved with BBB transport such as ATP1A2, SLC6A1, SLC1A2, and SLC1A4 and known astrocytic homeostasis-associated genes GLUL and NRXN1^25^ (Fig. 3d, Extended Data Fig. 3a). RHI-exposed samples also showed a significant reduction of homeostasis associated astrocytes, suggesting RHI elicits a phenotypic shift away from homeostatic function (Fig. 3b,c). Astro2 did not change in proportion across groups and showed the highest expression of GFAP, DPP10, and SLC38A1, a glutamate transporter suggestive of neuronal surveillance properties. Astrocytes with similar transcriptional profiles were previously characterized as “Layer 1 astrocytes”, suggesting a cortical layer localization for these cells^26^ (**b**).

Astro3 were enriched for RHI-exposed individuals (p = 0.005, Fig. 3b,c, Extended Data Fig. 3b). GO analysis identified “response to cytokine stimuli”, “inflammatory responses”, and “VEGFA signaling” terms (Extended Data Fig. 3a). Astro3 upregulated genes associated with astrocyte reactivity (CHI3L1, CD44, CLU, BCL6), inflammation (IL6R, IL1R1), and hypoxia (HIF1A, NRP1, ANGPTL4, Fig. 3d). CD44 and BCL6-positive astrocytes are observed in CTE white matter, suggesting common astrocytic responses across the grey and white matter in response to head trauma^7^. Astro4 made up only 2.9% of the total astrocytes and trended towards an increase in RHI-exposed individuals (p=0.07). This cluster transcriptionally resembled previously described disease associated astrocytes (DAA), expressing high CSMD1, a complement regulatory protein, along with cell-cell adhesion proteins DSCAM and bicarbonate regulator SLC4A4^27^. These findings support previous studies showing that astrocytes lose homeostatic function and upregulate inflammation, angiogenesis, and reactivity genes, and confirm the role of astrocytes in the early response to repetitive head trauma.

### Endothelial angiogenic response to RHI

**Extended Data Figure 4.**
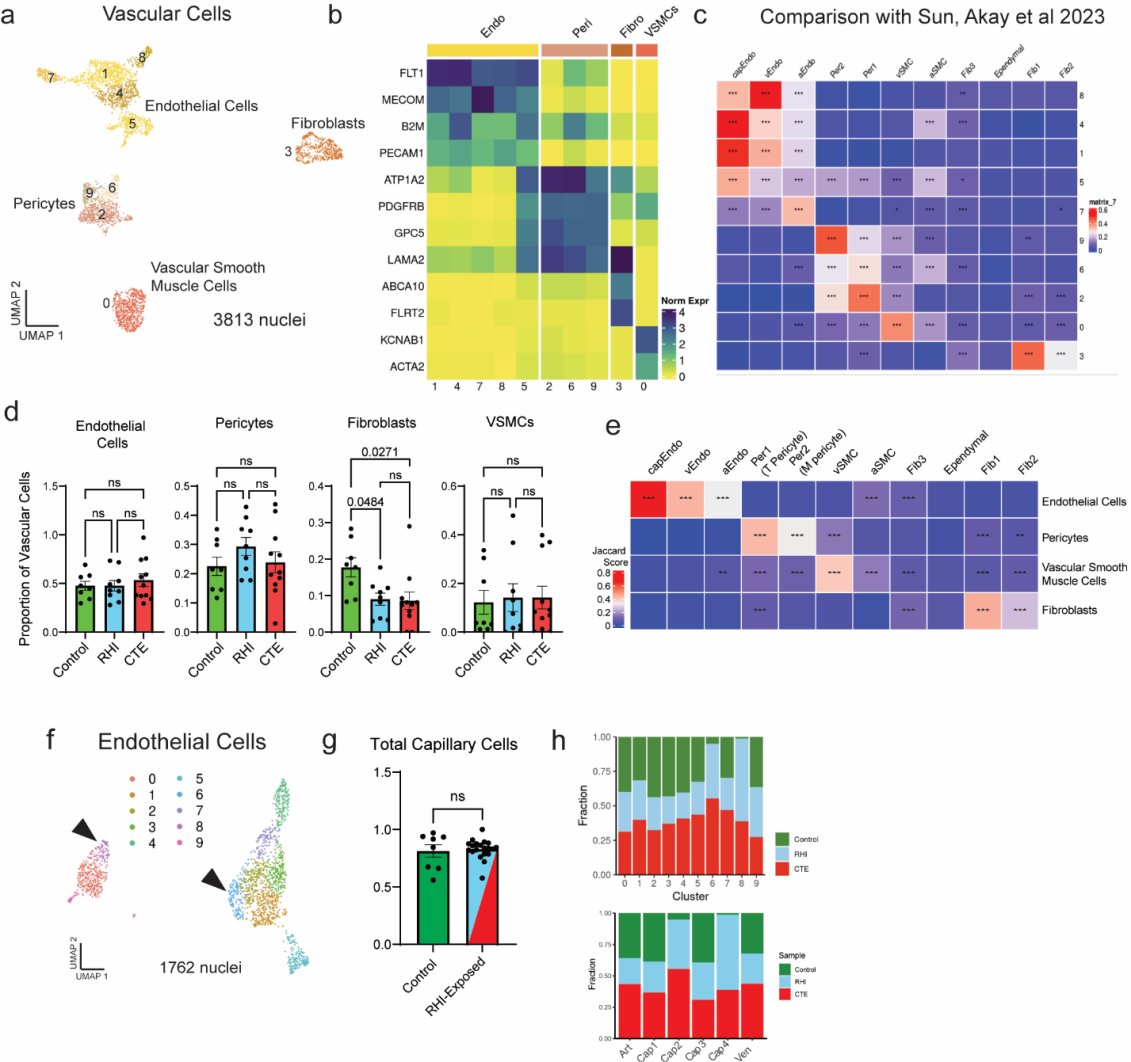
Vascular cell subtype identification and proportion analysis. **a.** UMAP showing all vascular cells colored by Seurat clustering. **b.** Heatmap depicting vascular cell marker expression. **c.** Heatmap depicting Jaccard scoring of vascular cell Seurat cluster DEGs compared to Sun and Akay et al. vascular subtype DEGs **, p<0.01, ***, p<0.001. **d.** Bar plots depicting pathological group proportions of vascular subtypes, bar represents mean, error bar represents standard error of the mean, dots represent individual samples. Statistical analysis performed by ANOVA with Bonferroni correction. **e.** Heatmap depicting Jaccard scoring of vascular cell subtype DEGs compared to Sun and Akay et al. vascular subtype DEGs **, p<0.01, ***, p<0.001. **f.** UMAP of endothelial cells colored by Seurat cluster, solid arrows indicate RHI and CTE enriched clusters. **g.** Bar plot depicting the fraction of capillary cells in control and RHI-exposed groups, dots represent individual samples, bar represents mean, error bars represent standard error of the mean. Statistical analysis performed by two sided Mann-Whitney U test. **h.** Stacked bar plot depicting the proportion of endothelial cell Seurat cluster (top) and subtype (bottom) distribution by pathological group.

Next, due to the key involvement of vascular dysfunction in CTE, we characterized the vascular response to RHI exposure^8,9^ (Extended Data Fig. 4a, b). Known cell type markers and comparison to published dataset markers were used to identify 1762 endothelial cells, 913 pericytes, 487 fibroblasts, and 651 vascular smooth muscle cells^28^ (Extended Data Fig. 4b,c,e). Only fibroblasts displayed significant changes in total proportion across pathological groups, decreasing from controls to RHI and CTE (p=0.048, 0.027, respectively, Extended Data Fig 4d). Endothelial cells were further labelled for arterial, venous, and capillary cells through comparison of expressed genes to published datasets^28^ (Extended Data Fig. 4c, e).

**Figure 4.**
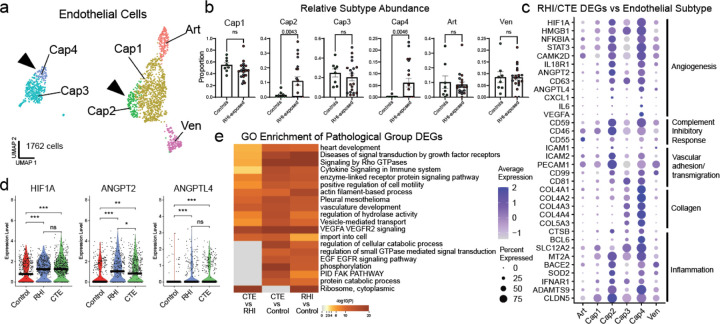
Endothelial angiogenic responses to RHI. **a.** UMAP of endothelial cells colored by endothelial cell subcluster. Solid arrows indicate RHI/CTE enriched clusters. **b.** Bar plots of relative endothelial subtype distribution across control and RHI-exposed samples, dots represent individual donors and are colored by pathological group identity. Bar indicates mean, error bars indicate standard error of the mean. Statistical analysis was performed by two-tailed Welch’s t-test or Mann-Whitney U test. **c.** Dot plot of selected upregulated RHI/CTE DEGs across endothelial subtypes annotated for function. **d.** Violin plots of Cap2/Cap4 DEGs, individual dots indicate individual cells, line indicates median. Plotting created with ggplot, statistics created with ggsignif. **, p <0.01, ***, p.<0.001. **e.** Annotated heatmap representing top GO terms identified in endothelial cell RHI and CTE DEGs.

To determine RHI and CTE-induced cell states, abundance analysis revealed that two populations of capillary cells, Cap2 and Cap4 were enriched for RHI and CTE samples (p = 0.004, 0.005, Fig. 4a,b). No differences were observed in the proportion of total capillary cells in RHI-exposed groups (Extended Data Fig 4j). Several canonical angiogenesis-associated genes such as HIF1A, ANGPT2, ANGPTL4, STAT3, CAMK2D, and NFKBID were significantly upregulated in Cap2 and Cap4 suggesting capillary cells in RHI-exposed groups may be responding to a local hypoxic environment (Fig. 4c,d). Three major complement regulatory proteins, CD59, CD55, and CD46, which inhibit complement-mediated cell lysis, were upregulated indicating a potential response to locally increased levels of complement (Fig. 4c). This is a protective mechanism also observed on cancerous angiogenic cells to evade complement mediated lysis^29^. Vascular adhesion and transmigration-associated genes ICAM1, ICAM2, PECAM1, and CD99 were increased in Cap2 and Cap4, indicating an increased potential for monocyte, T cell, neutrophil, or other peripheral cell entry across the endothelium (Fig. 4c). Peripheral macrophages are known to enter the parenchyma in CTE specifically at lesional vessels, likely through activated endothelium^30^. Cap4 also displayed high expression of collagen genes, a main feature of the BBB, suggesting a potential increase in collagen production as an attempt to repair damaged basement membranes (Fig. 4c). GO analysis identified VEGFA signaling, cytokine signaling, and vasculature development as significantly upregulated terms in RHI exposed endothelial cells (Fig. 4e).

Taken together, capillary cells undergo significant upregulation of angiogenesis and inflammation associated genes along with an increase in basement membrane components, identifying pathways that may underlie the known microvascular dysfunction in CTE^8,9^. Consistent with the changes observed in astrocytes and microglia following RHI, endothelial cell alterations are observed in individuals with RHI exposure without p-tau pathology, suggesting that microvascular alterations occur before the onset of neurodegeneration in CTE.

### Synaptic transcriptomic changes and loss of sulcal excitatory cortical layer 2/3 neurons

**Figure 5.**
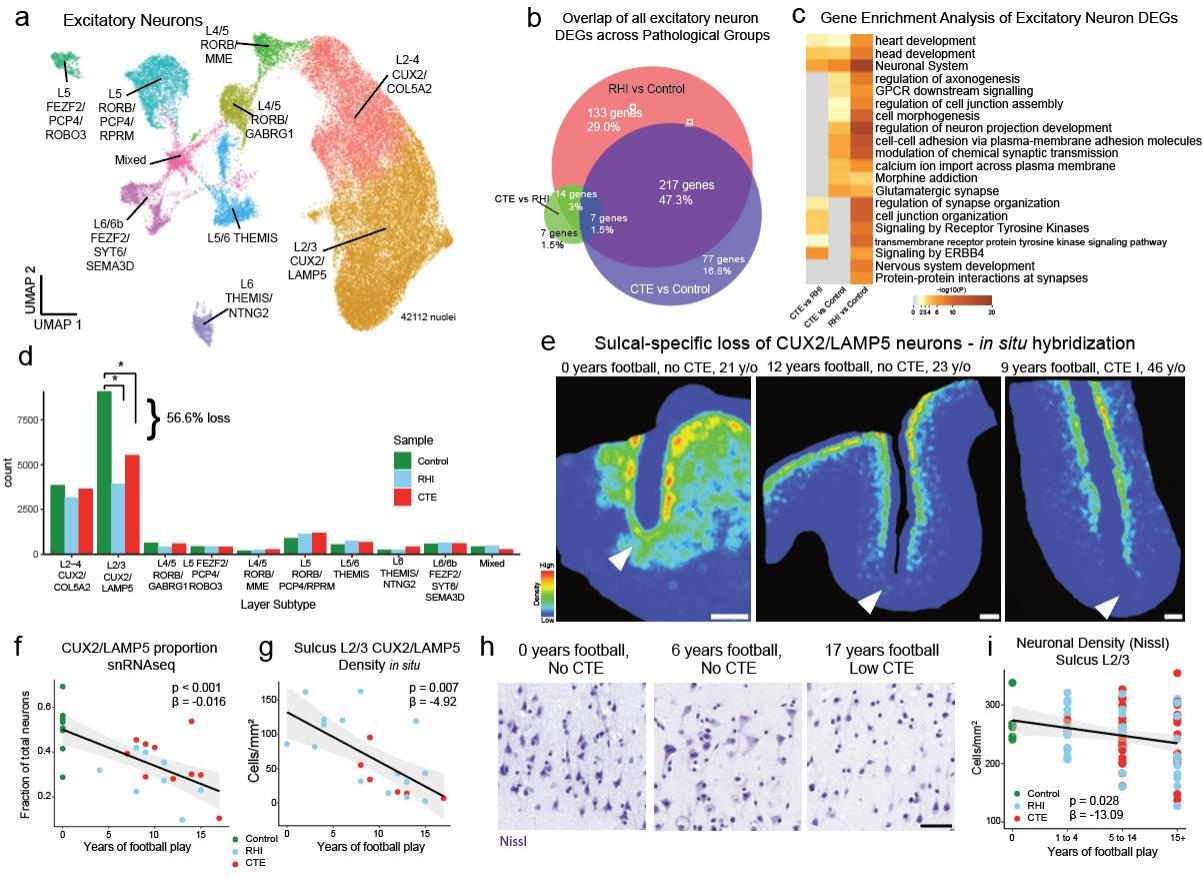
Synaptic transcriptomic changes and loss of sulcal excitatory layer 2/3 neurons. **a.** UMAP of excitatory neurons colored and labelled by layer subtype determined by expression of layer-specific markers. **b.** Venn Diagram depicting the overlap between DEGs from RHI vs Control, CTE vs Control, and RHI vs CTE comparisons. **c.** Heatmap of GO terms identified in comparisons listed in (b). **d.** Bar plot representing cell counts per pathological group for each excitatory neuron layer subtype. Statistical analysis performed by ordinary one-way ANOVA with Bonferroni correction. *, p<0.05. **e.** Representative density heatmap of CUX2/LAMP5 positive cells, solid arrows indicated depth of the cortical sulcus. Red indicates high cellular density; blue indicates low cellular density. Scale bar, 1 μm. **f.** Scatter plot showing the fraction of CUX2/LAMP5 neurons within total excitatory neurons in snRNAseq data against total years of football play colored by pathological group identity. Dots depict individual samples, line represents general linear model regression, grey shows 95% confidence interval **g.** Scatter plot showing cell density of CUX2/LAMP5 neurons in sulcal Layer 2/3 from *in situ* hybridization colored by pathological group identity compared to years of football play. Dots depict individual samples, line represents general linear model regression, grey shows 95% confidence interval. Statistics performed by general linear regression. **h.** Representative images of Nissl-stained neurons in superficial cortical layer 2/3, scale bar indicates 50 μm. **i.** Scatter plot showing Nissl-stained neuronal densities across football exposure groups. Dots depict individual samples, line represents general linear model regression, grey shows 95% confidence interval.

Next, due to the known dysfunction and degeneration of neurons and synaptic dysfunction following head trauma and in neurodegenerative disease, we examined neurons, labeling subclusters using known layer-specific markers^12,31–36^ (Fig. 5a, Supplementary Fig. 2e–j).

**Extended Data Figure 5.**
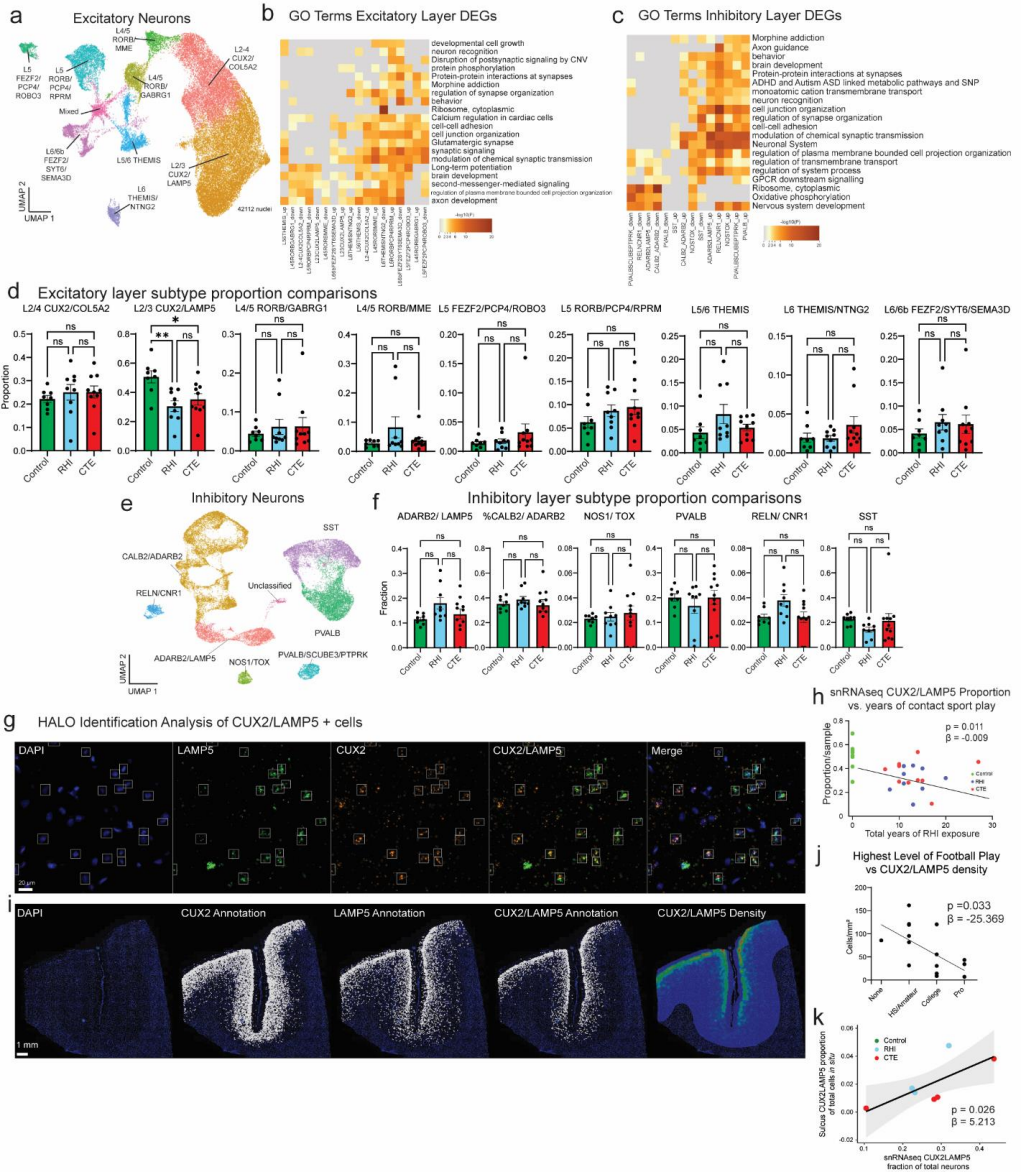
Neuron layer GO analysis, pathological group enrichment and RNAScope validation. **a.** UMAP depicting excitatory neurons colored by layer subtype. **b.** Heatmap showing GO analysis of excitatory layer up and downregulated DEGs. **c.** Heatmap showing GO analysis of inhibitory layer up and downregulated DEGs. **d.** Bar plots of excitatory neuron layer proportions by pathological group. Bar represents mean, dots represent individual samples, error bars show standard error of the mean. Statistical analysis performed by ANOVA with Bonferroni correction. *, p<0.05, **, p<0.01. **e.** UMAP showing inhibitory neurons colored by layer subtype. **f.** Bar plots of inhibitory neuron layer proportions by pathological group. Bar represents mean, dots represent individual samples, error bars show standard error of the mean. Statistical analysis performed by ANOVA with Bonferroni correction. **g,i.** Representative image showing RNAScope *in situ* hybridization of CUX2/LAMP5 image analysis at high power (g) with correct anatomical layer-wise distribution (i). **h.** Scatter plot of CUX2/LAMP5 proportion from snRNAseq against total years of football play colored by pathological group. Statistical analysis performed by linear regression, depicted as line. **j.** Scatter plot showing CUX2/LAMP5 density from *in situ* hybridization compared to highest level of football played. Statistical analysis performed by simple linear regression. Dots represent individual samples; line shows linear regression. **k.** Scatter plot of CUX2/LAMP5 cells identified by *in situ* experiment compared to proportion of CUX2/LAMP5 neurons from snRNAseq experiment. Statistical analysis performed by simple linear regression, depicted as line with 95% confidence intervals in grey.

47% of excitatory neuron DEGs were shared across RHI and CTE when compared to control and only 6% changed from RHI to CTE, suggesting that the greatest changes in excitatory neuronal transcriptional profiles occur with initial exposure to RHI (Fig. 5b). RHI-exposed groups were compared to controls and GO analysis of layer-specific DEGs demonstrated that “modulation of chemical synapses” and “cell-cell adhesion” processes were enriched in both analyses (Fig. 5c, Extended Data Fig. 5b). Genes associated with synaptic transmission such as SYN3, SNAP91, NRG1, HSP12A1 the Hsp70 gene, and extracellular matrix binding proteins such as CNTN5, CLSTN2 were upregulated across several excitatory neuron layers. Inhibitory neuron layer-wise DEGs displayed 40% fewer DEGs than excitatory neurons with only 184 DEGs specific to RHI-exposed groups compared to controls. GO analysis of inhibitory neuron layer specific DEGs showed common upregulation of synapse associated genes such as SYN3 and SYN2 and across layers and downregulation of GABA receptor gene GABRA1 (Extended Data Fig 5c). These data show that excitatory, and to a lesser extent inhibitory neurons undergo altered synaptic and cell adhesion gene expression following RHI suggesting potential alterations to the excitatory-inhibitory balance. Neuronal hyperexcitability is a known feature of early neurodegenerative processes and is accompanied by increased synaptic gene expression^37^. Additionally, cell adhesion molecules are involved in synapse remodeling, further supporting potential synaptic alterations in individuals with RHI exposure.

Since neurodegenerative processes and head trauma exposure can be associated with neuronal loss, we investigated layer-specific cell composition in RHI-exposed groups. No pathological group enrichment was found in inhibitory neurons (Extended Data Fig 5e,f). However, differential abundance analysis of excitatory Layer 2/3 CUX2/LAMP5 neurons demonstrated a significant decrease in individuals with a history of RHI, regardless of disease status (Fig. 5d, Extended Data Fig. 5d). These results were confirmed via multinomial dirichlet multinomial regression to account for the compositional nature of snRNAseq data^13^. Individuals with an average of 12.7 years of RHI exposure had an average of 56% fewer CUX2/LAMP5 neurons than age-matched unexposed controls and this loss was associated with the number of years of playing American football or, in the few cases with other types of contact sports play, total years of RHI exposure, independent of age at death (p<0.001, Fig. 5f, Extended Data Fig. 5h). To validate and expand on the snRNAseq, quantitative histology with RNAScope *in situ* hybridization showed that reduced CUX2/LAMP5+ neuron density was associated with greater years of football play (p = 0.007, β = −4.92) and highest level of football played (p = 0.033, β = −25.34, Fig 5e,g, Extended Data Fig. 5g,j,i). Examining cases that were included in both *in situ* hybridization and snRNAseq methods, cellular proportions of CUX2/LAMP5 neurons significantly correlated (p =0.02, Extended Data Fig. 5k). CUX2/LAMP5+ cell loss was most evident at the depth of the cortical sulcus, a region highly affected by RHI-induced damage and CTE pathology^38^ (Fig. 5e). To confirm the association between years of football play and neuronal loss, total neuronal densities were determined using Nissl staining of 86 young individuals with 0–28 years of American football play. Individuals were grouped by 0, 1–4, 5–14, and 15+ years of football play based on previously defined thresholds for CTE risk^3^. Layer 2/3 sulcal neuronal density significantly decreased with increased binned years of football play independent of age at death (Fig. 5h,i, p = 0.028, β =−13.09). No correlation between neuronal loss and p-tau deposition was observed (p = 0.387 *in situ*, p = 0.825 Nissl), suggesting neuronal loss occurs prior to and independent of pathologic protein deposition in early stages of disease. Overall, these results show the first evidence that exposure to RHI alone might drive significant neuronal loss and disfunction, which helps explain early symptom onset in young athletes without the presence of significant p-tau pathology.

## Discussion

In this study we utilize a combination of single nucleus RNA sequencing, multiplex *in situ* hybridization, and immunohistological analyses to describe and validate a unique dataset of young individuals with exposure to RHI. We describe distinct microglial subsets that emerge following RHI and persist with CTE, correlate with years of contact sport play, and associate with p-tau pathology. We demonstrate that RHI induces early robust phenotypic changes in astrocytes and endothelial cells. We also show alterations to synaptic and cell adhesion gene expression across neuronal subtypes. Finally, we observed a sulcus-specific loss of cortical layer 2/3 neurons that correlated with exposure to RHI prior to p-tau deposition.

Microglial, astrocytic, and endothelial cell transcriptomic subtypes have been described in several neurodegenerative diseases and in severe traumatic brain injury, however this is the first study to demonstrate these changes in a young of a cohort with exposure to repeated non-concussive head impacts. Interestingly, hypoxia-associated changes are present across these three cell types, suggesting an important role for vascular dysfunction and bolstering previous evidence of vascular remodeling in CTE. Forces from head trauma disproportionately affect blood vessels, causing a lasting endothelial response affecting blood brain barrier integrity and oxygen delivery in affected regions^38^. Activated endothelium, local hypoxia, and a breached BBB may trigger a feedback loop activating astrocytes and microglia with each head impact. Repeated blows to the head in short succession likely reactivate an already inflamed system, disallowing sufficient time for full repair, and preventing a return to homeostasis. Through this repetitive reactivation, the inflammatory response becomes self-sustaining and chronic, the mechanisms of which remain unclear. This is substantiated by the increased microglial activation observed decades after retirement from contact sports and found to correlate with the number of years of RHI exposure in this study and others^5^.

Oligodendrocytes and OPCs were not observed significantly affected by RHI exposure. Previous work from our group has shown that oligodendrocytes and OPCs in the white matter of older individuals with CTE are lost and display altered transcriptional phenotypes with disease^7^. Oligodendrocyte and OPC loss may be a specific white matter alteration, or a change occurring at later stages of disease.

Finally, we observed a marked ~56% decrease in superficial layer 2/3 excitatory neurons in RHI-exposed individuals at the depths of the cortical sulci, the region known to sustain the most mechanical force upon head trauma, and the initial region of p-tau accumulation in CTE^38^. This is the first study to demonstrate such a dramatic loss of a specific neuronal subtype in young individuals solely driven by RHI exposure. This is especially concerning considering several of the observed individuals had no neuropathologic protein deposition, suggesting neurodegeneration might begin sooner than CTE onset. A recent study also showed cortical thinning in frontal regions of high school football players, suggesting our observed findings might explain these imaging related studies^39^. Neuronal loss might also explain symptoms of traumatic encephalopathy syndrome, the clinical criteria for antemortem CTE diagnosis, in young athletes^2,40,41^. Layer 2/3 neurons make cortico-cortical connections and, in the frontal cortex, are associated with depressive behaviors and moderation of stress^42^. Interestingly, layer 2/3 neurons have been shown to be vulnerable in other neurodegenerative and psychiatric disorders and are susceptible to p-tau accumulation in AD ^12,32^. Therefore, one may speculate that superficial layer 2/3 excitatory neurons are highly susceptible to damage regardless of source and our data captured the loss across a range of RHI doses. Although RHI damage is driving the early neuronal loss, it is likely that as p-tau deposition becomes more severe, neuronal death and dysfunction will become more related to pathogenic protein accumulation observed in other diseases such as AD. The complex interplay of neuronal death mechanisms represents an important area of study to better understand how different treatment strategies and biomarkers can have unique and separate effects in early versus late disease.

One limitation of our study is the small amount of tissue in each sample. CTE is an inherently patchy disease and diagnosis is made based on the presence of a pathognomonic CTE lesion consisting of a focus of perivascular p-tau accumulation at the depth of the cortical sulcus. It is therefore possible that sampling may have missed regions of important cellular responses. Future studies of RHI-exposed individuals should aim to sample from several areas of the brain to improve detection of cellular responses. Additionally, due to the inherent difficulties in acquiring non-disease, non-RHI exposed young postmortem human samples, some control cases included in the Nissl quantification were female which may complicate direct comparisons to male athletes. However, no statistical correlation was found when sex was compared to neuronal densities.

In conclusion, these results highlight the growing concerns linked to long term RHI exposure from contact sports. The data presented here is some of the first direct evidence that demonstrates RHI-driven cellular perturbations occur prior to the development of CTE and can be observed in young individuals, many with no obvious brain pathology. Novel biomarkers and therapeutic interventions will be vital in identifying the early changes observed in contact sport athletes prior to developing neurodegeneration.

## Methods

### Neuropathological Diagnosis

Brain tissue was obtained from the CTE and the National Center for PTSD Brain Banks. Identical intake and tissue processing procedures occur with both brain banks. Four controls included in Nissl quantification were provided by the Iowa Neurobank Core. Neuropathological examination was performed by board certified neuropathologists as described previously^10,43^. Diagnosis of CTE was determined using published consensus criteria^10,43^. Demographics such as athletic history, military history, traumatic brain injury history, and RHI history were queried during telephone interview with next of kin as detailed previously^10,43^. Institutional review board approval for brain donation was obtained through the Boston University Alzheimer’s Disease and CTE Center, Human Subjects Institutional Review Board of the Boston University School of Medicine, and VA Bedford Healthcare System (Bedford, MA). Individuals were included in the study based on frozen tissue availability, quality (RIN>4), and diagnosis. Exclusion criteria included neuropathological diagnosis other than CTE, moderate to severe traumatic brain injury directly prior to death, age of death greater than 51 or less than 25. Control cases did not have exposure to any RHI, were negative for any neurodegenerative disease, and did not carry any diagnosis of a neuropsychological disorder.

### Single Nucleus RNA Sequencing

Fresh frozen brain tissue was collected from the dorsolateral frontal cortex of each donor at the depth of the cortical sulcus. Visual delineation of grey/white matter was used to collect 50μg of tissue. Nuclei isolation and sorting were performed on two donor samples per day, randomizing for diagnosis and age. Tissue was kept on ice throughout nuclei isolation. Tissue was homogenized and lysed in NST Buffer with DAPI (146mM NaCl, 10mM Tris, 1mM CaCl2, 21mM MGCl2, 0.1%BSA, 0.1% NP-40, 40U/ml Protector RNase Inhibitor, DAPI) and snipped with scissors on ice for 10 minutes. Debris was removed using a 70μm filter. Cells were spun down and resuspended in nuclei storage buffer (2% BSA, 400U/mL Protector RNase Inhibitor) to reach a concentration of 500–1000 nuclei/μL. Nuclei were purified for DAPI positive cells with a FACS-Aria flow cytometer to remove debris and processed using the Chromium Next GEM Single Cell 3’ Reagents Kit V2 (10x Genomics) to create cDNA libraries. Samples were pooled in two batches sequenced with Azenta to a read depth of 30,000 reads/cell on an Illumina NovaSeq.

### Processing, Quality Control, and Clustering of Single Nucleus RNA Sequencing Data

CellRanger v6.0.1 was used to align reads to the GRCH38 reference and generate filtered count matrices containing 233,555 across all samples. The “runCellQC” function in the *singleCellTK* R package was used to generate quality control metrics and doublet calls^44,45^. Contamination from ambient RNA was identified using decontx using the full raw matrix as the “background” for each sample^46^. Nuclei were removed if they had ambient RNA contamination fraction greater than 0.3, mitochondrial or ribosomal percentage greater than 5%, total counts less than 750, or genes detected less than 500. The Seurat workflow within the *singleCellTK* package was used for clustering starting with the decontaminated counts from decontX^47^. Briefly, the data was normalized and scaled using runSeuratNormalizeData and runSeuratScaleData. Highly variable genes were identified using runSeuratFindHVG with method ‘vst’. Principle components were determined using runSeuratPCA. UMAP dimensionality reduction was calculated using runSeuratUMAP. Clusters across all cell types were identified using the runSeuratFindClusters function at a resolution of 0.3. After initial clustering all the cells, clusters that were predominantly doublets (>50%) were removed and produced the final dataset of 170,717 nuclei (Extended Data Fig. 1h–k). All GO analysis was performed using MetaScape default settings^48^. DEG lists for all comparisons available in Supplementary Tables 6–16.

### Cell type identification

Cell type markers verified by previous human snRNAseq studies were used to identify clusters that belonged to individual cell types (Extended Data Fig. 1m,n). Cell types were subsetted out using subsetSCEColData and reclustered by the same Seurat method described above with the addition of running Harmony to account for sample-to-sample variability^49^. Clusters expressing high levels of >1 cell type marker were removed. Excitatory and inhibitory neurons identified from the full dataset were clustered together to determine neuronal subtypes. Four clusters (1, 2, 19, 21) were found to express low levels of neuronal genes and astrocytic genes (SLC1A2, SLC1A3), and were single-batch enriched (80–90%) therefore these clusters were not included in downstream analysis (Supplementary Fig. 2a–d).

### Histological Tissue Processing

Formalin fixed, paraffin embedded tissue was sectioned and labelled as previously described^50^. Briefly, 10μm sections were allowed to dry, baked, dewaxed, and rehydrated prior to antibody labelling. For immunofluorescent staining, epitope retrieval was performed using a pH6 or pH9 buffer and boiling for 15 minutes in the microwave. Sections were blocked for 30 minutes at room temperature with 3% donkey serum and primary antibodies (Supplementary Table 3,4) were conjugated for 1 hour at room temperature. Secondary antibodies were conjugated for 30 minutes, and TSA dyes were incubated for 10 minutes. Slides were coverslipped with ProLong Gold Antifade mounting medium (Invitrogen) and imaged at 40x on a Vectra Polaris whole-slide scanner. Images were spectrally unmixed using inForm software prior to image analysis. For Nissl staining, sections were hydrated and stained in 0.01% thionin for 20–40 seconds and dehydrated back to xylene before coverslipping in Permount mounting media and imaging on an Aperio GT450 scanner at 40x.

### Single molecule fluorescent mRNA in situ hybridization and IHC codetection

Tissue was embedded in Optimal Cutting Temperature medium (Sakura Tissue-Tek) and was brought to cryostat temperature (−20° C) before cutting. Chuck temperature was raised to −12°/ −10°C for optimal cutting conditions. Tissue was sectioned at 16 µm thickness onto Fisher SuperFrost slides. Direction of tissue orientation relative to the depth of the cortical sulcus was randomized across samples. Sections were fixed in cold 4°C 10% Neutral Buffered Formalin for 60 minutes and dehydrated in 50%, 70%, 100%, and 100% ethanol for 5 minutes each at room temperature. Fluorescent *in situ* hybridization was performed using RNAScope kits (Advanced Cell Diagnostics) optimized on the Leica BOND Rx automated slide staining system. Slides were pretreated with protease for 15 minutes. Opal TSA dyes were used for visualization at a concentration of 1:300–500. A positive and negative control probe was run for each block before staining with targeted probes. For immunohistochemical codetection of p-tau, sections were run through the RNAScope protocol as described and then manually stained with the immunohistochemical protocol described in the Histological Analysis section.

### Image Analysis

Analysis of fluorescent *in situ* hybridization (FISH) was performed using the HALO FISH v3.2.3 algorithm and analysis of IHC protein staining was performed using the Object Colocalization v2.1.4 algorithm. Analysis parameters were kept consistent across samples. Analysis of Nissl staining was performed using HALO (Indica Laboratory) Nuclei Segmentation AI algorithm. Neurons were selected for training based on previously published criteria^51^. Briefly, the classifier was given examples of parenchyma annotated for neurons which were considered cells with a Nissl-positive cytoplasm and a visible nucleus (Supplementary Fig. 3a). Nissl+ densities across batches were not significantly different and statistical tests of Nissl densities were corrected for staining batch. For FISH and Nissl sections, the depth of the cortical sulcus was defined and annotated as the bottom third of a gyral crest and sulcus pair. Layer 2/3 was annotated using layer-specific FISH markers or for Nissl stains by an expert observer.

### Statistics

Analyses were performed using GraphPad Prism 10 and SPSS v.29. Dirichlet multinomial regression was used to test for cell type and excitatory neuron cell type enrichment using the scCoda v0.1.9 Python package^13^. Comparisons across the three pathological groups were performed using ANOVA with Bonferroni correction or Brown Forsyth with Dunnett post-hoc test. Comparison across control and RHI-exposed groups was performed with a t-test with Welch correction or Mann Whitney U test. Comparison of *in situ* hybridization analysis was performed using linear regression, p-tau burden was normalized using log10 transformation of positive area density. Nissl staining was assessed using linear regression and correcting for age at death, and staining batch. Jaccard similarity scoring was performed using the GeneOverlap package by comparing lists of DEGs. All DEGs were filtered by a log2fc of 0.15 and FDR of <0.05. Years of football play was used as a variable for exposure instead of total years of play which includes exposure from all sports played because it was a more consistent predictor of cellular changes.

## Extended Data

## Supplementary Material

Supplement 1 Supplementary Figure 1. Cell type proportions, OPCs, and Oligodendrocytes. **a.** Bar plots of overall cell type proportions across pathological groups with each dot representing a sample, bars represent the mean, error bars represent standard error of the mean. Statistical analysis performed by ANOVA with Bonferroni correction. **b.** UMAP depicting OPCs colored by Seurat clustering, solid arrow indicating RHI/CTE depleted cluster. **c.** Stacked bar plot showing OPC Seurat cluster distribution across pathological groups. **d.** Bar plots showing OPC cluster distribution across control and pathological group or control and RHI-exposed samples, bar represents mean, error bars show standard error of the mean. Statistical analysis performed by ANOVA with Bonferroni correction (left) and two-tailed Mann-Whitney U test. **e.** Heatmap showing GO analysis of OPC cluster DEGs. **f.** UMAP showing oligodendrocytes colored by Seurat cluster, solid arrow indicates RHI and CTE depleted cluster. **g.** Stacked bar plot showing oligodendrocyte pathological group distribution per Seurat cluster. **h.** Bar plots representing cluster distribution across pathological groups or control and RHI-exposed samples. Bar represents mean, error bar represents standard error of the mean. Statistical analysis performed by ANOVA with Bonferroni correction (left) or two-tailed t-test (right). **i.** Heatmap showing GO analysis of oligodendrocyte cluster DEGs. **j.** UMAP showing T cells colored by Seurat cluster. **k.** Heatmap of GO analysis of T cell cluster DEGs.

Supplement 2 Supplementary Figure 2. Neuronal layer subtype identification. **a.** UMAP depicting all neurons clustered together colored by Seurat cluster. **b.** Dotplot of gene expression of inhibitory and excitatory neuron and astrocyte marker genes Seurat clusters from (a). **c.** UMAP from (a) colored by cell type determination. **d.** Stacked bar plot of sequencing batch distribution of Seurat clusters from (a). **e.** UMAP showing excitatory neurons colored by Seurat cluster. **f.** UMAP showing excitatory neurons colored by later subtype. **g.** Dotplot showing expression of excitatory neuron layer subtype genes in excitatory neuron Seurat clusters from (e). **h.** UMAP showing inhibitory neurons colored by Seurat cluster. **i.** UMAP showing inhibitory neurons colored by layer subtype. **j.** Dotplot showing expression of inhibitory neuron layer subtype genes across inhibitory neuron Seurat clusters from (h).

Supplement 3 Supplementary Figure 3. Nissl-positive Neuron Identification with HALO AI. **a.** Representative image depicting layer 2/3 neurons stained with Nissl histological stain (left), and corresponding AI identification of neurons in green. Scale bar indicates 50 μm.

Supplement 4 Supplementary Table 1. Demographics Summary of SnRNAseq Samples. Table showing a summary of the demographic information for the snRNAseq samples. Data expressed as mean ± standard deviation. Age at death and years of exposure analyzed with one-way ANOVA.

Supplement 5 Supplementary Table 2. Demographics table of SnRNAseq Samples. Table showing demographic information from samples included in snRNAseq dataset. PMI = post-mortem interval.

Supplement 6 Supplementary Table 3. Demographics table of *in situ* hybridization and Nissl Samples. **a.** Table showing demographic information from samples included in *in situ* hybridization experiments. **b.** Table showing demographic information from samples included in Nissl staining experiments. PMI = post-mortem interval.

Supplement 7 Supplementary Table 4. List of Antibodies.

Supplement 8 Supplementary Table 5. List of *In Situ* probes.

Supplement 9 Supplementary Table 6. DEGs of All Cells in snRNAseq Dataset. **a,b.** List of differentially expressed genes in respective clusters depicted in Figure 1c and Extended Data Figure 1l. Log2_FC = log-2 fold change. FDR = false discovery rate.

Supplement 10 Supplementary Table 7. DEGs of Excitatory and Inhibitory Neurons in snRNAseq Dataset. **a.** List of differentially expressed genes in Seurat clusters of inhibitory and excitatory neuron clusters depicted in Supplementary Figure 2a. Log2_FC = log-2 fold change. FDR = false discovery rate.

Supplement 11 Supplementary Table 8. DEGs of Vascular Cells in snRNAseq Dataset. **a.** List of differentially expressed genes in clusters depicted in Extended Data Figure 4a. Log2_FC = log-2 fold change. FDR = false discovery rate.

Supplement 12 Supplementary Table 9. DEGs of Microglia in snRNAseq Dataset. **a.** List of differentially expressed genes in microglial subtype clusters depicted in Figure 2a. **b-e.** List of differentially expressed genes in microglial pathological group comparisons. **f.** List of genes significantly differentially expressed across pseudotime depicted in Figure 2l. Log2_FC = log-2 fold change. FDR = false discovery rate.

Supplement 13 Supplementary Table 10. DEGs of Astrocytes in snRNAseq Dataset. **a.** List of differentially expressed genes in astrocyte subtype clusters depicted in Figure 4a. **b-e.** List of differentially expressed genes in endothelial pathological group comparisons. Log2_FC = log-2 fold change. FDR = false discovery rate.

Supplement 14 Supplementary Table 11. DEGs of Endothelial Cells in snRNAseq Dataset. **a.** List of differentially expressed genes in endothelial subtype clusters depicted in Figure 3a. **b-e.** List of differentially expressed genes in astrocyte pathological group comparisons. Log2_FC = log-2 fold change. FDR = false discovery rate.

Supplement 15 Supplementary Table 12. DEGs of Oligodendrocytes in snRNAseq Dataset. **a.** List of differentially expressed genes in oligodendrocyte Seurat clusters depicted in Supplementary Figure 1b. **b-e.** List of differentially expressed genes in oligodendrocyte pathological group comparisons. Log2_FC = log-2 fold change. FDR = false discovery rate.

Supplement 16 Supplementary Table 13. DEGs of Oligodendrocyte Precursor Cells in snRNAseq Dataset. **a.** List of differentially expressed genes in oligodendrocyte Seurat clusters depicted in Supplementary Figure 1f. **b-e.** List of differentially expressed genes in oligodendrocyte precursor cell pathological group comparisons. Log2_FC = log-2 fold change. FDR = false discovery rate.

Supplement 17 Supplementary Table 14. DEGs of T Cells in snRNAseq Dataset. **a.** List of differentially expressed genes in T Cell Seurat clusters depicted in Supplementary Figure 1j. Log2_FC = log-2 fold change. FDR = false discovery rate.

Supplement 18 Supplementary Table 15. DEGs of Excitatory Neurons in snRNAseq Dataset. **a.** List of differentially expressed genes in excitatory neuron subtype clusters depicted in Figure 5a. **b-d.** List of differentially expressed genes in excitatory neuron pathological group comparisons **e-n.** List of differentially expressed genes in individual excitatory neuron subtype pathological group comparisons. Log2_FC = log-2 fold change. FDR = false discovery rate.

Supplement 19 Supplementary Table 16. DEGs of Inhibitory Neurons in snRNAseq Dataset. **a.** List of differentially expressed genes in inhibitory neuron subtype clusters depicted in Supplementary Figure 2i. **b-d.** List of differentially expressed genes in inhibitory neuron pathological group comparisons **e-l.** List of differentially expressed genes in individual inhibitory neuron subtype pathological group comparisons. Log2_FC = log-2 fold change. FDR = false discovery rate.
