# Supplementary figures and images for: Repetitive head impacts induce neuronal loss and neuroinflammation in young athletes
Source: bioRxiv. 2025 Feb 10:2024.03.26.586815. Originally published 2024 Mar 28. Preprint. [Version 2] doi: 10.1101/2024.03.26.586815 (PMC10996668; doi:10.1101/2024.03.26.586815)

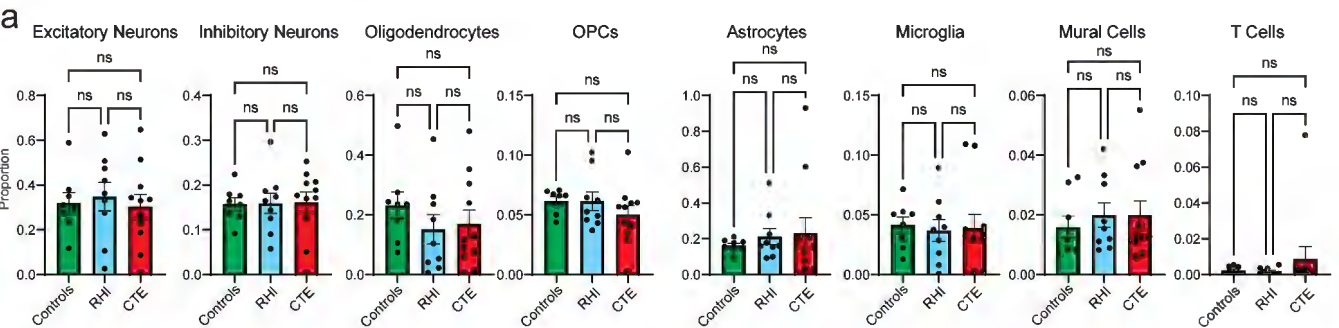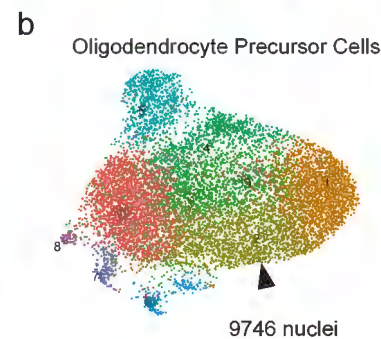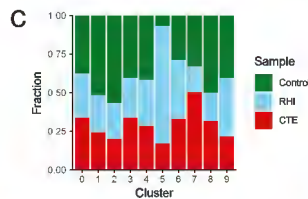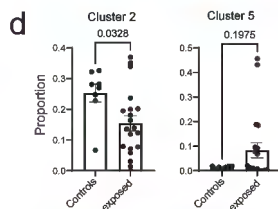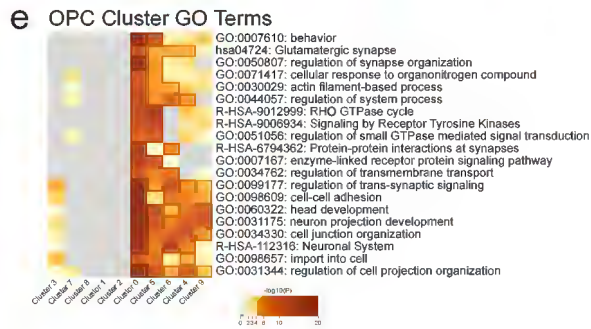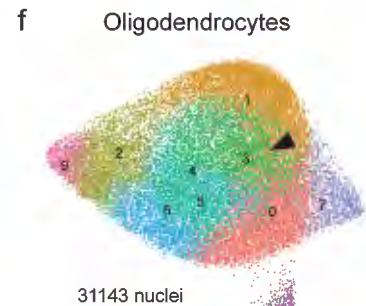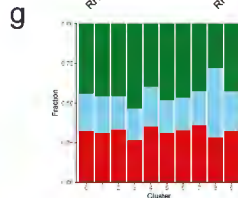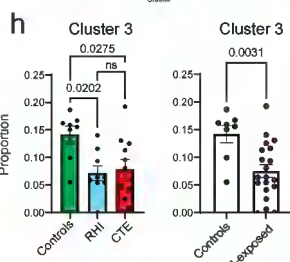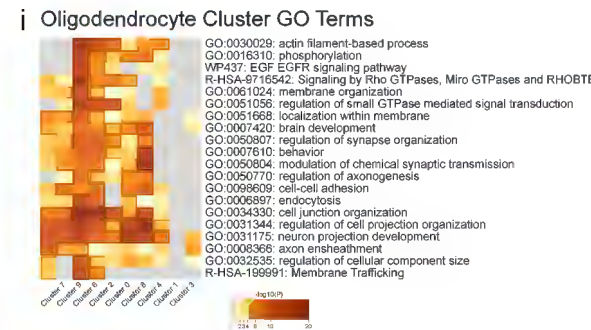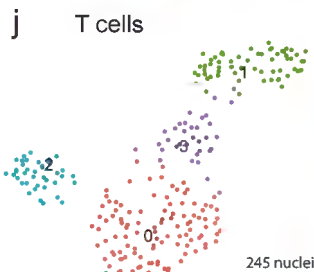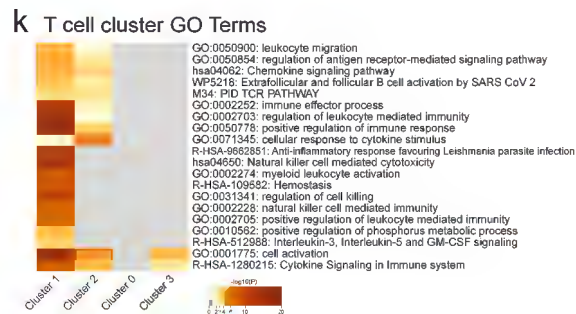

Supplement: Supplement 1 — Supplementary Figure 1. Cell type proportions, OPCs, and Oligodendrocytes. a. Bar plots of overall cell type proportions across pathological groups with each dot representing a sample, bars represent the mean, error bars represent standard error of the mean. Statistical analysis performed by ANOVA with Bonferroni correction. b. UMAP depicting OPCs colored by Seurat clustering, solid arrow indicating RHI/CTE depleted cluster. c. Stacked bar plot showing OPC Seurat cluster distribution across pathological groups. d. Bar plots showing OPC cluster distribution across control and pathological group or control and RHI-exposed samples, bar represents mean, error bars show standard error of the mean. Statistical analysis performed by ANOVA with Bonferroni correction (left) and two-tailed Mann-Whitney U test. e. Heatmap showing GO analysis of OPC cluster DEGs. f. UMAP showing oligodendrocytes colored by Seurat cluster, solid arrow indicates RHI and CTE depleted cluster. g. Stacked bar plot showing oligodendrocyte pathological group distribution per Seurat cluster. h. Bar plots representing cluster distribution across pathological groups or control and RHI-exposed samples. Bar represents mean, error bar represents standard error of the mean. Statistical analysis performed by ANOVA with Bonferroni correction (left) or two-tailed t-test (right). i. Heatmap showing GO analysis of oligodendrocyte cluster DEGs. j. UMAP showing T cells colored by Seurat cluster. k. Heatmap of GO analysis of T cell cluster DEGs. [file media-1.pdf]

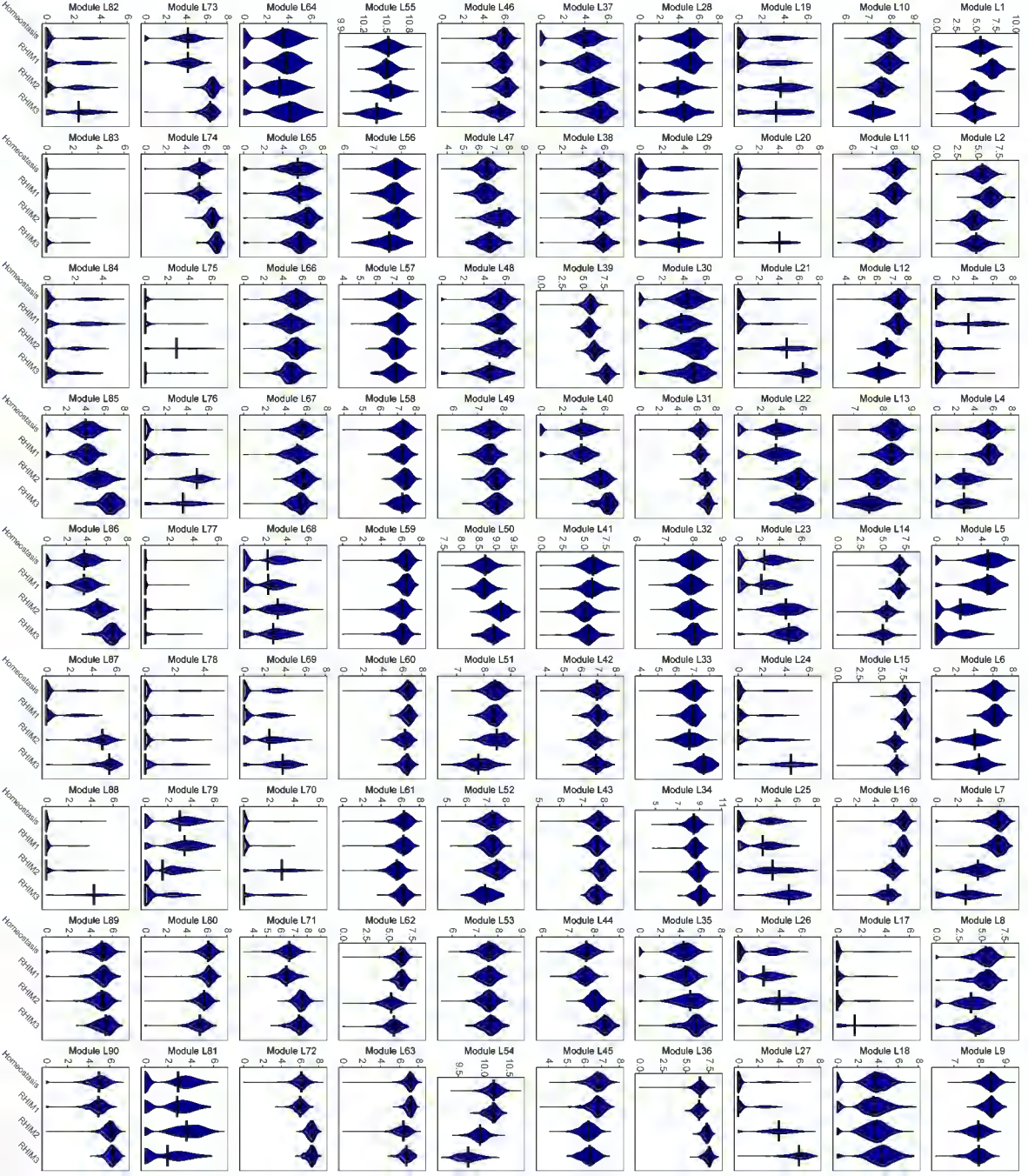

Supplement: Supplement 3 — Supplementary Figure 3. Microglia Celda Modules. Violin plots depicting Celda module expression for modules 1-90 across Homeostatic, RHIM1, RHIM2, and RHIM3 microglial clusters. Black bar is the median statistic from ggsignif. [file media-3.pdf]

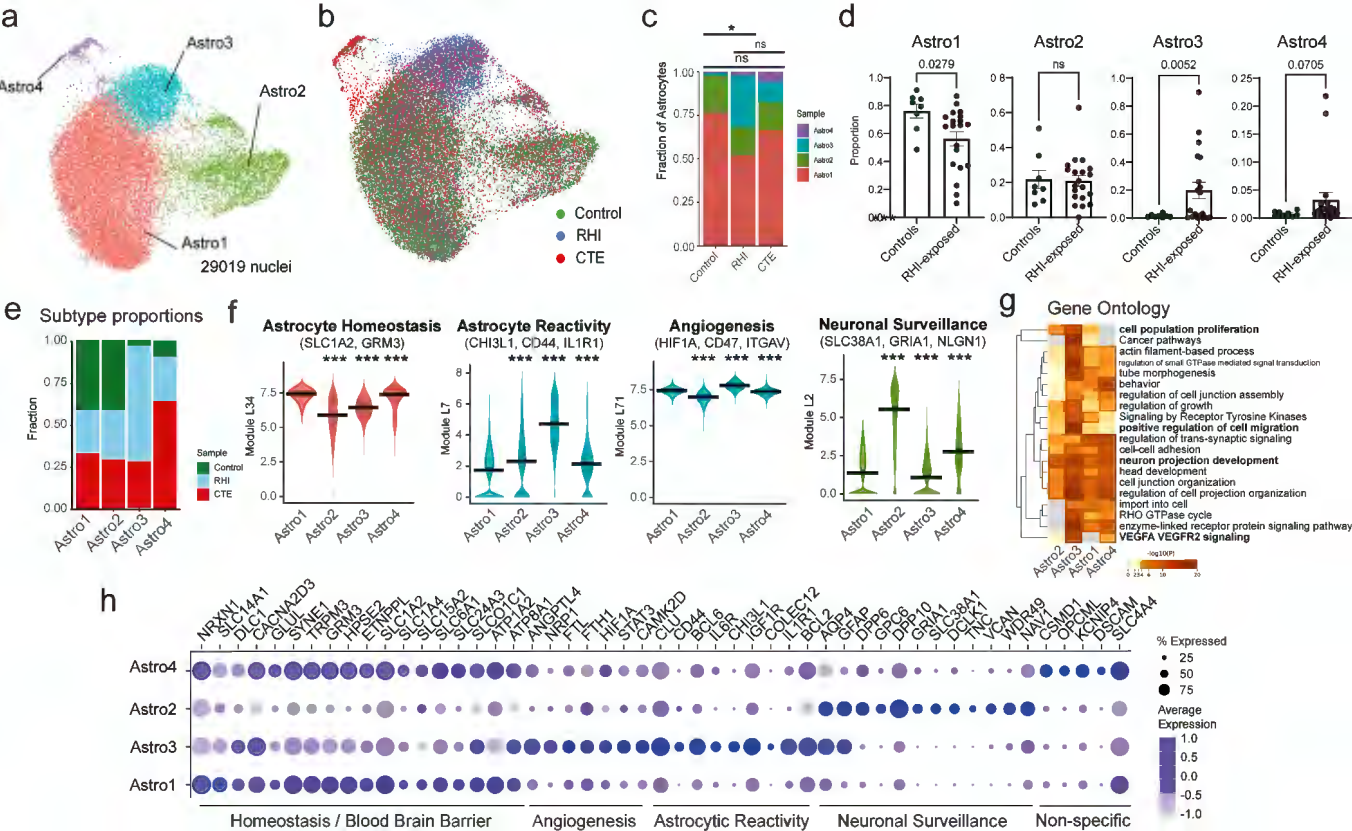

Supplement: Supplement 5 — Supplementary Figure 5. Astrocytic responses to head trauma. a. UMAP representing 4 astrocytic subtypes. b. UMAP from (a) colored by pathological group. c. Stacked bar plots showing astrocyte subtype distribution across pathological groups, statistics performed by chi-squared test. d. Bar plots showing astrocyte subcluster distribution in control and RHI-exposed samples, dots represent individual donors colored by pathological group identity. Bars represent mean, error bars represent standard error of the mean. Statistical analysis was performed using two-tailed Mann Whitney U-test. e. Stacked bar plots showing pathological distribution across astrocyte subtypes. f. Violin plots showing Celda module expression across astrocyte subtypes. Black bar showing median statistic. Colored by astrocyte subtype most associated with specific module expression. Statistical analysis performed by linear mixed effects model. g. Gene ontology analysis of astrocytic subtypes performed by Metascape. h. Dot plot representing expression of selected DEGs across astrocytic subtype and annotated by function. [file media-5.pdf]

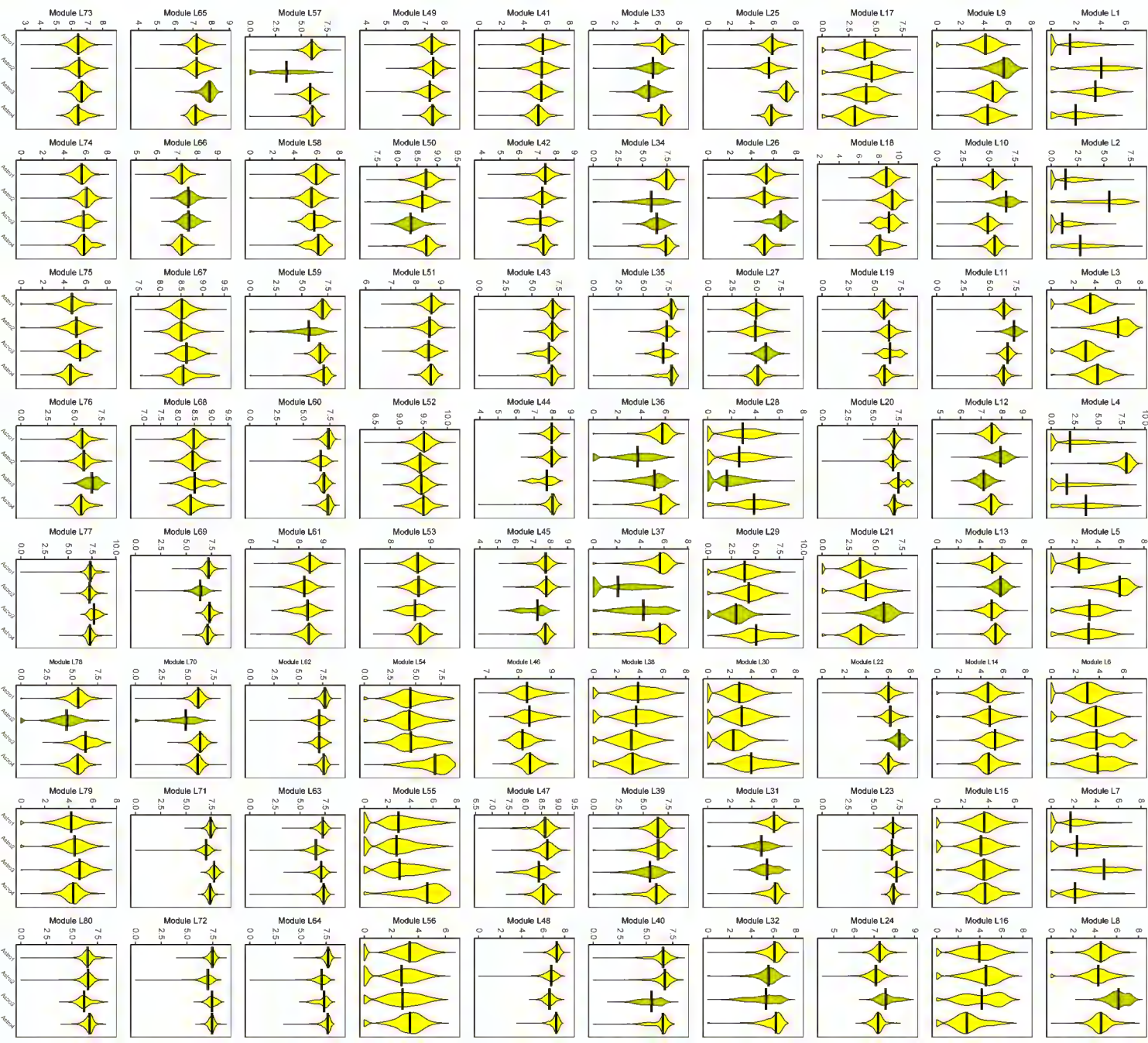

Supplement: Supplement 6 — Supplementary Figure 6. Astrocyte Celda Modules. Violin plots depicting Celda module expression for modules 1–80 across Astro1, Astro2, Astro3, and Astro4 astrocyte clusters. Black bar is the median statistic from ggsignif. [file media-6.pdf]

# Endothelial Cell Modules L1- L60

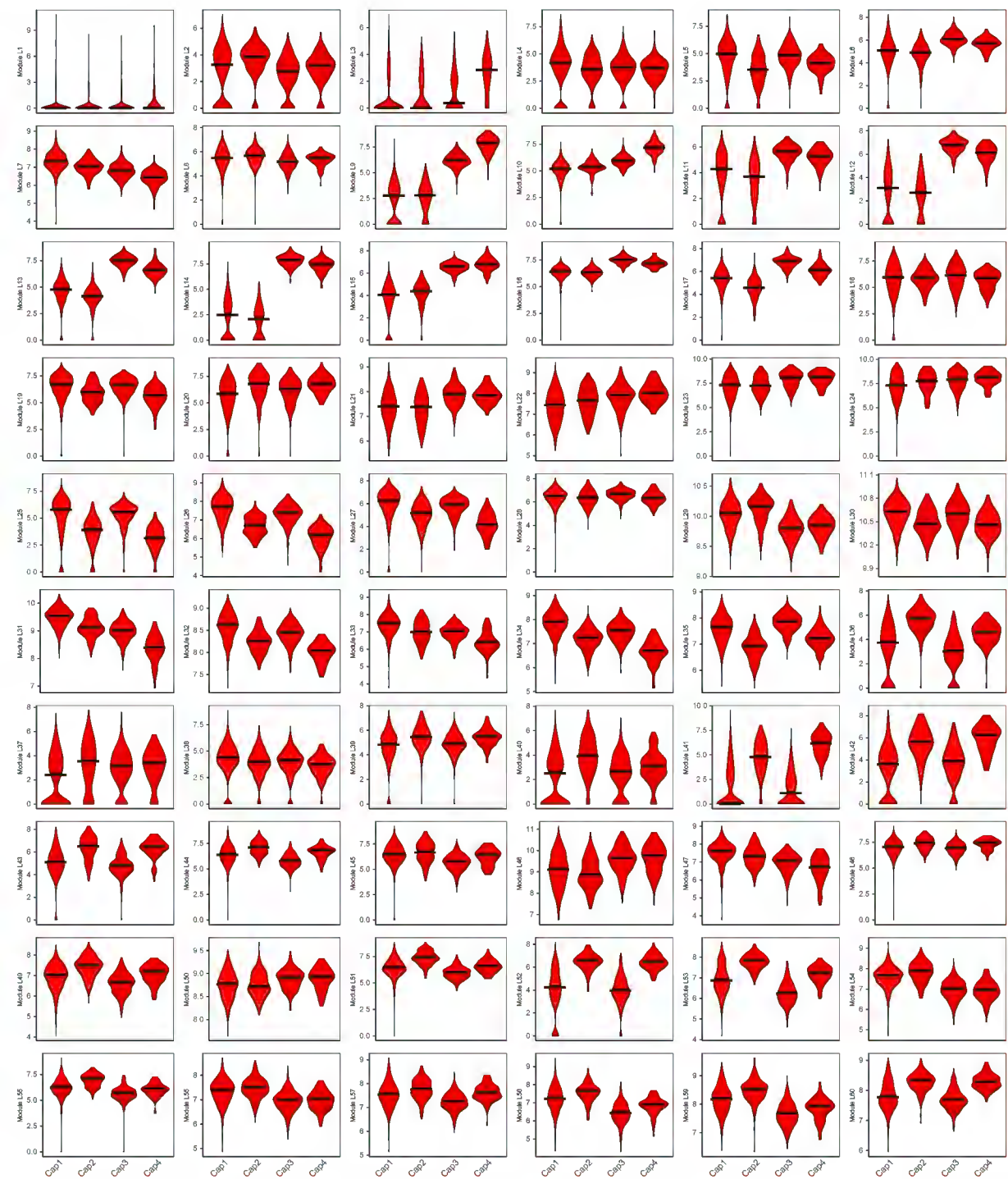

Supplement: Supplement 7 — Supplementary Figure 7. Endothelial Cell Celda Modules. Violin plots depicting Celda module expression for modules 1–60 across Cap1, Cap2, Cap3, and Cap4 endothelial cell clusters. Black bar is the median statistic from ggsignif. [file media-7.pdf]

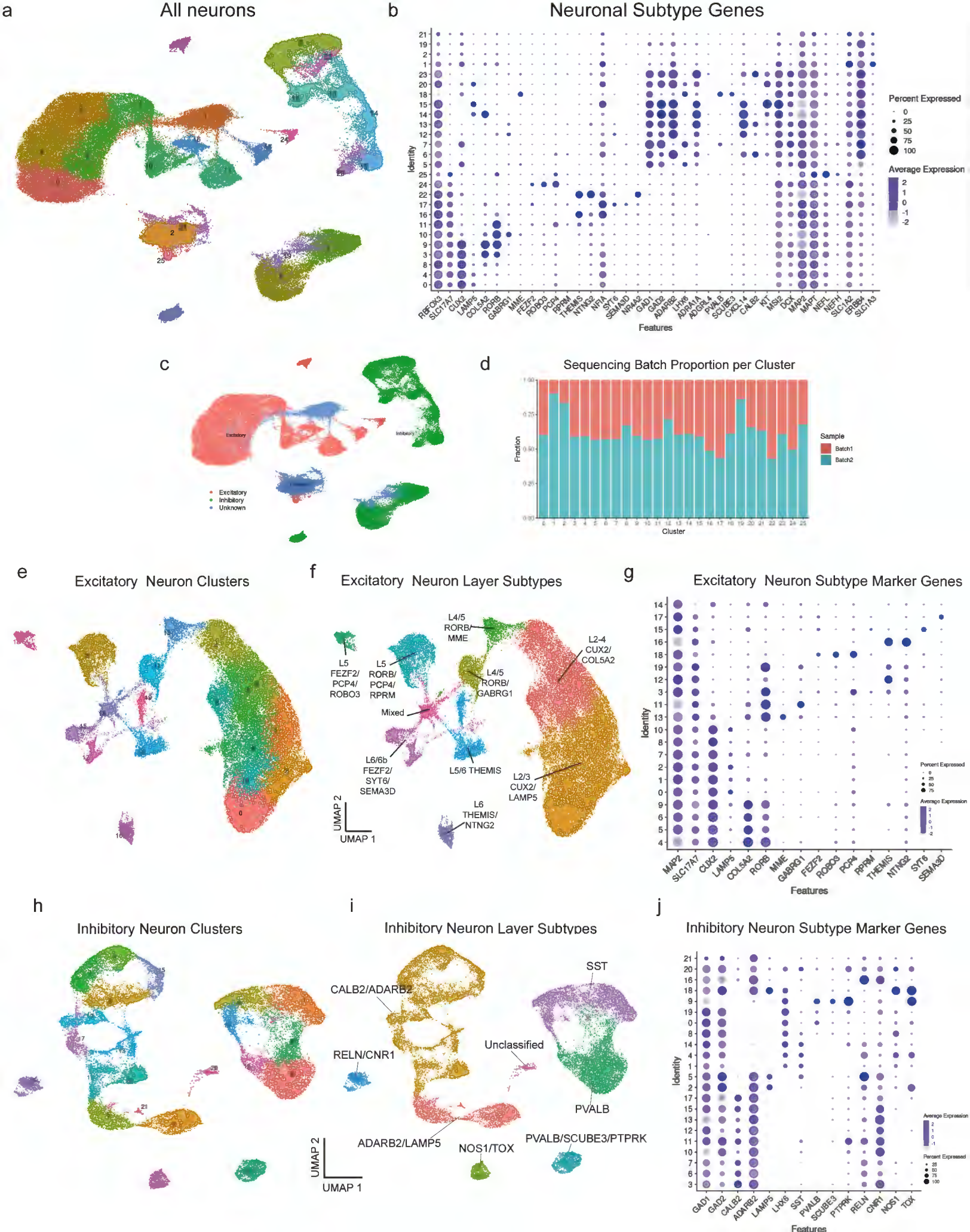

Supplement: Supplement 8 — Supplementary Figure 8. Neuronal layer subtype identification. a. UMAP depicting all neurons clustered together colored by Seurat cluster. b. Dot plot of gene expression of inhibitory and excitatory neuron and astrocyte marker genes Seurat clusters from (a). c. UMAP from (a) colored by cell type determination. d. Stacked bar plot of sequencing batch distribution of Seurat clusters from (a). e. UMAP showing excitatory neurons colored by Seurat cluster. f. UMAP showing excitatory neurons colored by later subtype. g. Dot plot showing expression of excitatory neuron layer subtype genes in excitatory neuron Seurat clusters from (e). h. UMAP showing inhibitory neurons colored by Seurat cluster. i. UMAP showing inhibitory neurons colored by layer subtype. j. Dot plot showing expression of inhibitory neuron layer subtype genes across inhibitory neuron Seurat clusters from (h). [file media-8.pdf]
