# Supplementary material for: Repetitive head impacts induce neuronal loss and neuroinflammation in young athletes
Source: bioRxiv. 2025 Feb 10:2024.03.26.586815. Originally published 2024 Mar 28. Preprint. [Version 2] doi: 10.1101/2024.03.26.586815 (PMC10996668; doi:10.1101/2024.03.26.586815)
Supplement: Supplement 4 — Supplementary Figure 4. Cellular subtype metadata correlations and Microglia external dataset projection. a. Correlation heatmap of selected metadata and cellular subtypes. Heatmap color depicts and direction and magnitude of Pearson’s r correlation value. Statistical analysis performed by Pearson correlation. *, p <0.05, **, p < 0.01, ***, p < 0.001. b. UMAP of combined and reclustered microglia from Sun et al 2023 dataset and current dataset. Left, colored by microglial subtypes from Sun data, left colored by subtypes in current dataset. [file media-4.pdf]

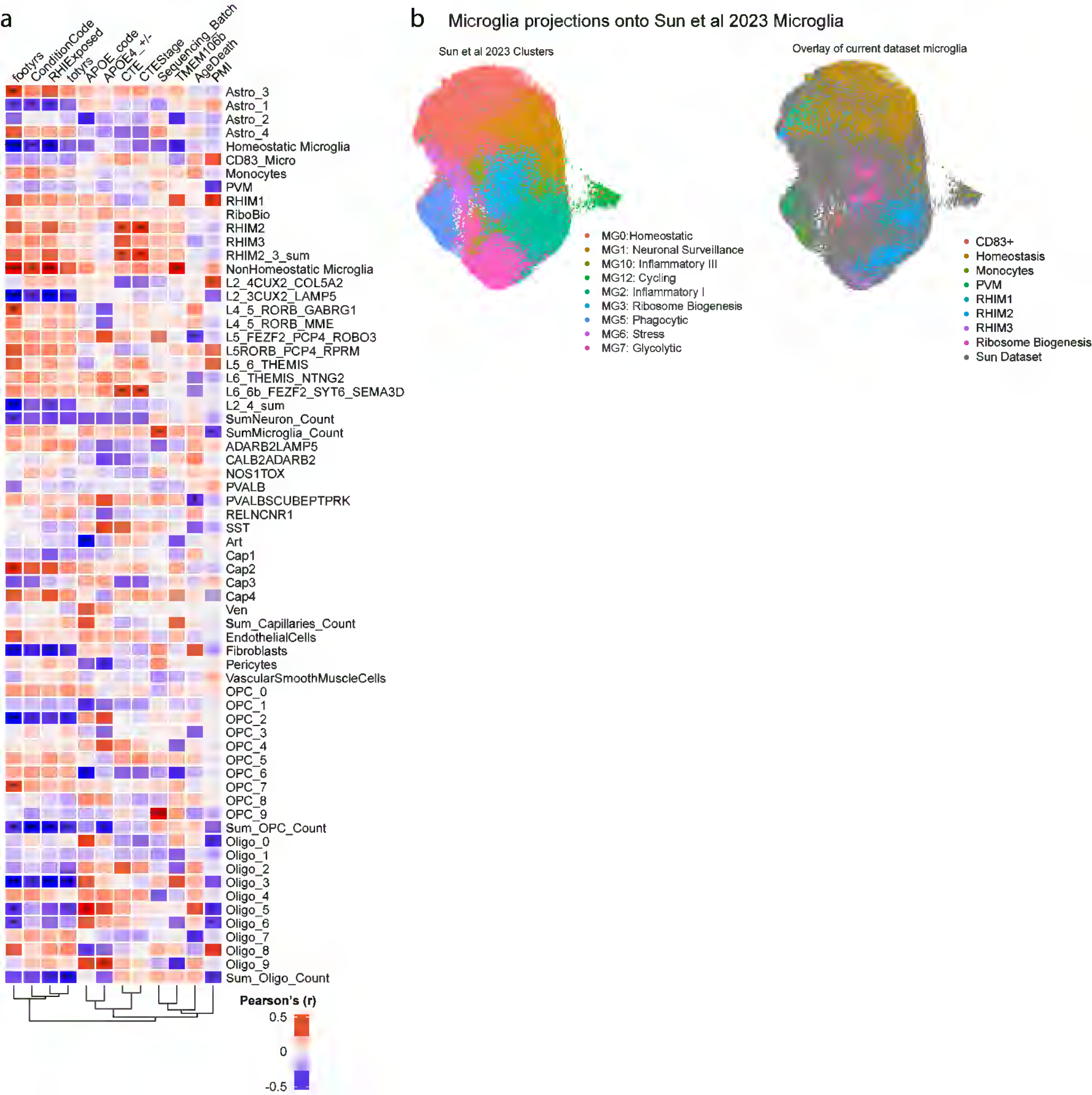

a
footyrs
ConditionCode
RHIExposed
totyrs
APOE_code
APOE4_+/-
CTE
CTEStage
Sequencing_Batch
TMEM106b
AgeDeath
PMI
Astro_3
Astro_1
Astro_2
Astro_4
Homeostatic Microglia
CD83_Micro
Monocytes
PVM
RHIM1
RiboBio
RHIM2
RHIM3
RHIM2_3_sum
NonHomeostatic Microglia
L2_4CUX2_COL5A2
L2_3CUX2_LAMP5
L4_5_RORB_GABRG1
L4_5_RORB_MME
L5_FEZF2_PCP4_ROBO3
L5RORB_PCP4_RPRM
L5_6_THEMIS
L6_THEMIS_NTNG2
L6_6b_FEZF2_SYT6_SEMA3D
L2_4_sum
SumNeuron_Count
SumMicroglia_Count
ADARB2LAMP5
CALB2ADARB2
NOS1TOX
PVALB
PVALBSCUBEPTPRK
RELNCNR1
SST
Art
Cap1
Cap2
Cap3
Cap4
Ven
Sum_Capillaries_Count
EndothelialCells
Fibroblasts
Pericytes
VascularSmoothMuscleCells
OPC_0
OPC_1
OPC_2
OPC_3
OPC_4
OPC_5
OPC_6
OPC_7
OPC_8
OPC_9
Sum_OPC_Count
Oligo_0
Oligo_1
Oligo_2
Oligo_3
Oligo_4
Oligo_5
Oligo_6
Oligo_7
Oligo_8
Oligo_9
Sum_Oligo_Count
Pearson's (r)
0.5
0
-0.5
b
Microglia projections onto Sun et al 2023 Microglia
Sun et al 2023 Clusters
MG0:Homeostatic
MG1: Neuronal Surveillance
MG10: Inflammatory III
MG12: Cycling
MG2: Inflammatory I
MG3: Ribosome Biogenesis
MG5: Phagocytic
MG6: Stress
MG7: Glycolytic
Overlay of current dataset microglia
CD83+
Homeostasis
Monocytes
PVM
RHIM1
RHIM2
RHIM3
Ribosome Biogenesis
Sun Dataset
