# Supplementary material for: Repetitive head impacts induce neuronal loss and neuroinflammation in young athletes
Source: bioRxiv. 2025 Feb 10:2024.03.26.586815. Originally published 2024 Mar 28. Preprint. [Version 2] doi: 10.1101/2024.03.26.586815 (PMC10996668; doi:10.1101/2024.03.26.586815)
Supplement: Supplement 9 — Supplementary Figure 9. Neuron layer GO analysis, pathological group enrichment and RNAScope validation. a. UMAP depicting excitatory neurons colored by layer subtype. b. Heatmap showing GO analysis of excitatory layer up and downregulated DEGs. c. Heatmap showing GO analysis of inhibitory layer up and downregulated DEGs. d. Bar plots of excitatory neuron layer proportions by pathological group. Bar represents mean, dots represent individual samples, error bars show standard error of the mean. Statistical analysis performed by ANOVA with Bonferroni correction. *, p<0.05, **, p<0.01. e. UMAP showing inhibitory neurons colored by layer subtype. f. Bar plots of inhibitory neuron layer proportions by pathological group. Bar represents mean, dots represent individual samples, error bars show standard error of the mean. Statistical analysis performed by ANOVA with Bonferroni correction. g. Representative image showing RNAScope in situ hybridization of CUX2/LAMP5 image analysis with correct anatomical layer-wise distribution. g. Representative image of CUX2/LAMP5 in situ with white squares showing HALO identification of double-positive cells. [file media-9.pdf]

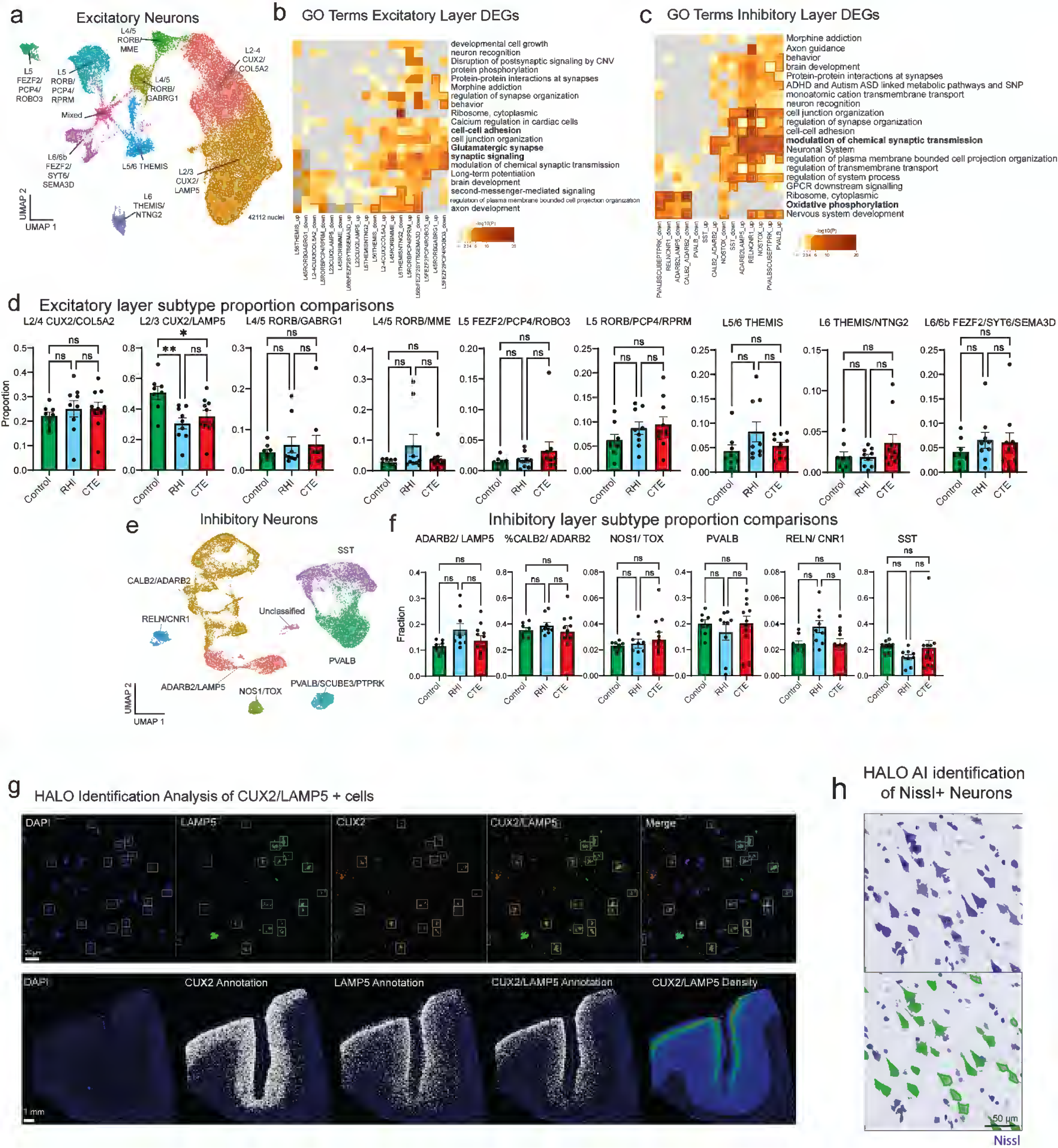
a
Excitatory Neurons
L5 FEZF2/ PCP4/ ROBO3
L5 RORB/ PCP4/ RPRM
L4/5 RORB/ MME
L4/5 RORB/ GABRG1
L2-4 CUX2/ COL5A2
Mixed
L6/6b FEZF2/ SYT6/ SEMA3D
L5/6 THEMIS
L2/3 CUX2/ LAMP5
L6 THEMIS/ NTNG2
42112 nuclei
UMAP 2
UMAP 1
b
GO Terms Excitatory Layer DEGs
developmental cell growth
neuron recognition
Disruption of postsynaptic signaling by CNV
protein phosphorylation
Protein-protein interactions at synapses
Morphine addiction
regulation of synapse organization
behavior
Ribosome, cytoplasmic
Calcium regulation in cardiac cells
cell-cell adhesion
cell junction organization
Glutamatergic synapse
synaptic signaling
modulation of chemical synaptic transmission
Long-term potentiation
brain development
second-messenger-mediated signaling
regulation of plasma membrane bounded cell projection organization
axon development
-log10(P)
c
GO Terms Inhibitory Layer DEGs
Morphine addiction
Axon guidance
behavior
brain development
Protein-protein interactions at synapses
ADHD and Autism ASD linked metabolic pathways and SNP
monoatomic cation transmembrane transport
neuron recognition
cell junction organization
regulation of synapse organization
cell-cell adhesion
modulation of chemical synaptic transmission
Neuronal System
regulation of plasma membrane bounded cell projection organization
regulation of transmembrane transport
regulation of system process
GPCR downstream signalling
Ribosome, cytoplasmic
Oxidative phosphorylation
Nervous system development
-log10(P)
d
Excitatory layer subtype proportion comparisons
L2/4 CUX2/COL5A2
L2/3 CUX2/LAMP5
L4/5 RORB/GABRG1
L4/5 RORB/MME
L5 FEZF2/PCP4/ROBO3
L5 RORB/PCP4/RPRM
L5/6 THEMIS
L6 THEMIS/NTNG2
L6/6b FEZF2/SYT6/SEMA3D
Proportion
ns
*
**
Control
RHI
CTE
e
Inhibitory Neurons
SST
CALB2/ADARB2
RELN/CNR1
Unclassified
PVALB
ADARB2/LAMP5
NOS1/TOX
PVALB/SCUBE3/PTPRK
UMAP 2
UMAP 1
f
Inhibitory layer subtype proportion comparisons
ADARB2/ LAMP5
%CALB2/ ADARB2
NOS1/ TOX
PVALB
RELN/ CNR1
SST
Fraction
ns
Control
RHI
CTE
g
HALO Identification Analysis of CUX2/LAMP5 + cells
DAPI
LAMP5
CUX2
CUX2/LAMP5
Merge
DAPI
CUX2 Annotation
LAMP5 Annotation
CUX2/LAMP5 Annotation
CUX2/LAMP5 Density
1 mm
h
HALO AI identification of Nissl+ Neurons
50 μm
Nissl
