# Supplementary material for: Repetitive head impacts induce neuronal loss and neuroinflammation in young athletes
Source: bioRxiv. 2025 Feb 10:2024.03.26.586815. Originally published 2024 Mar 28. Preprint. [Version 2] doi: 10.1101/2024.03.26.586815 (PMC10996668; doi:10.1101/2024.03.26.586815)
Supplement: Supplement 2 — Supplementary Figure 2. Celda module workflow and cell type expression. a. Celda module workflow diagram. b. Examples of Celda module expression in microglia. UMAPs show module expression, heatmaps show per-cell expression with genes listed on the right. Genes can also be viewed in Supplementary Table 17. c. Examples of Celda module expression in endothelial cells. UMAPs show module expression, heatmaps show per-cell expression with genes listed on the right. Genes can also be viewed in Supplementary Table 18. [file media-2.pdf]

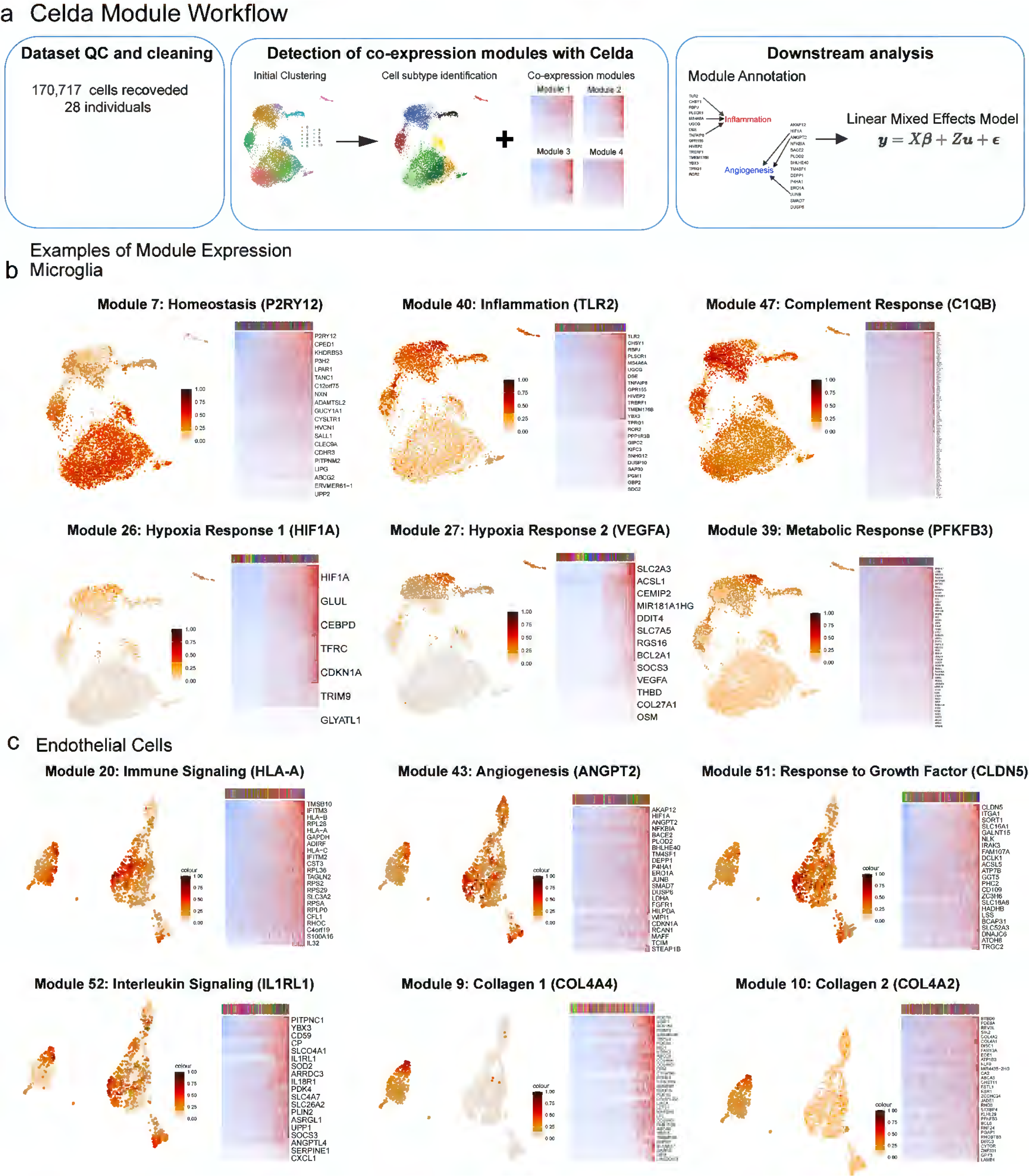
a Celda Module Workflow
Dataset QC and cleaning
170,717 cells recoveded
28 individuals
Detection of co-expression modules with Celda
Initial Clustering
Cell subtype identification
Co-expression modules
Module 1
Module 2
Module 3
Module 4
Downstream analysis
Module Annotation
Inflammation
Angiogenesis
Linear Mixed Effects Model
$y = X\beta + Zu + \epsilon$
Examples of Module Expression
b Microglia
Module 7: Homeostasis (P2RY12)
P2RY12
CPED1
KHDRBS3
P3H2
LPAR1
TANC1
C12orf75
NXN
ADAMTSL2
GUCY1A1
CYSLTR1
HVCN1
SALL1
CLEC9A
CDHR3
PITPNM2
LIPG
ABCG2
ERVMER61-1
UPP2
Module 40: Inflammation (TLR2)
TLR2
CHSY1
RBPJ
PLSCR1
MS4A6A
UGCG
DSE
TNFAIP8
GPR155
HIVEP2
TRERF1
TMEM176B
YBX3
TPRG1
ROR2
PPP1R3B
GIPC2
KIFC3
SNHG12
DUSP10
SAP30
PGM1
GBP2
SDC2
Module 47: Complement Response (C1QB)
Module 26: Hypoxia Response 1 (HIF1A)
HIF1A
GLUL
CEBPD
TFRC
CDKN1A
TRIM9
GLYATL1
Module 27: Hypoxia Response 2 (VEGFA)
SLC2A3
ACSL1
CEMIP2
MIR181A1HG
DDIT4
SLC7A5
RGS16
BCL2A1
SOCS3
VEGFA
THBD
COL27A1
OSM
Module 39: Metabolic Response (PFKFB3)
c Endothelial Cells
Module 20: Immune Signaling (HLA-A)
TMSB10
IFITM3
HLA-B
RPL28
HLA-A
GAPDH
ADIRF
HLA-C
IFITM2
CST3
RPL36
TAGLN2
RPS2
RPS29
SLC3A2
RPSA
RPLP0
CFL1
RHOC
C4orf19
S100A16
IL32
Module 43: Angiogenesis (ANGPT2)
AKAP12
HIF1A
ANGPT2
NFKBIA
BACE2
PLOD2
BHLHE40
TM4SF1
DEPP1
P4HA1
ERO1A
JUNB
SMAD7
DUSP6
LDHA
FGFR1
HILPDA
WIPI1
CDKN1A
RCAN1
MAFF
TCIM
STEAP1B
Module 51: Response to Growth Factor (CLDN5)
CLDN5
ITGA1
SORT1
SLC16A1
GALNT15
NLK
IRAK3
FAM107A
DCLK1
ACSL5
ATP7B
GGT5
PHC2
CD109
ZC3H6
SLC18A8
HADHB
LSS
BCAP31
SLC52A3
DNAJC6
ATOH8
TRGC2
Module 52: Interleukin Signaling (IL1RL1)
PITPNC1
YBX3
CD59
CP
SLCO4A1
IL1RL1
SOD2
ARRDC3
IL18R1
PDK4
SLC4A7
SLC26A2
PLIN2
ASRGL1
UPP1
SOCS3
ANGPTL4
SERPINE1
CXCL1
Module 9: Collagen 1 (COL4A4)
Module 10: Collagen 2 (COL4A2)
