# Supplementary material for: Repetitive head impacts induce neuronal loss and neuroinflammation in young athletes
Source: bioRxiv. 2024 Mar 28:2024.03.26.586815. Preprint. [Version 1] doi: 10.1101/2024.03.26.586815 (PMC10996668; doi:10.1101/2024.03.26.586815)
Supplement: Supplementary file 1 [file media-1.pdf]

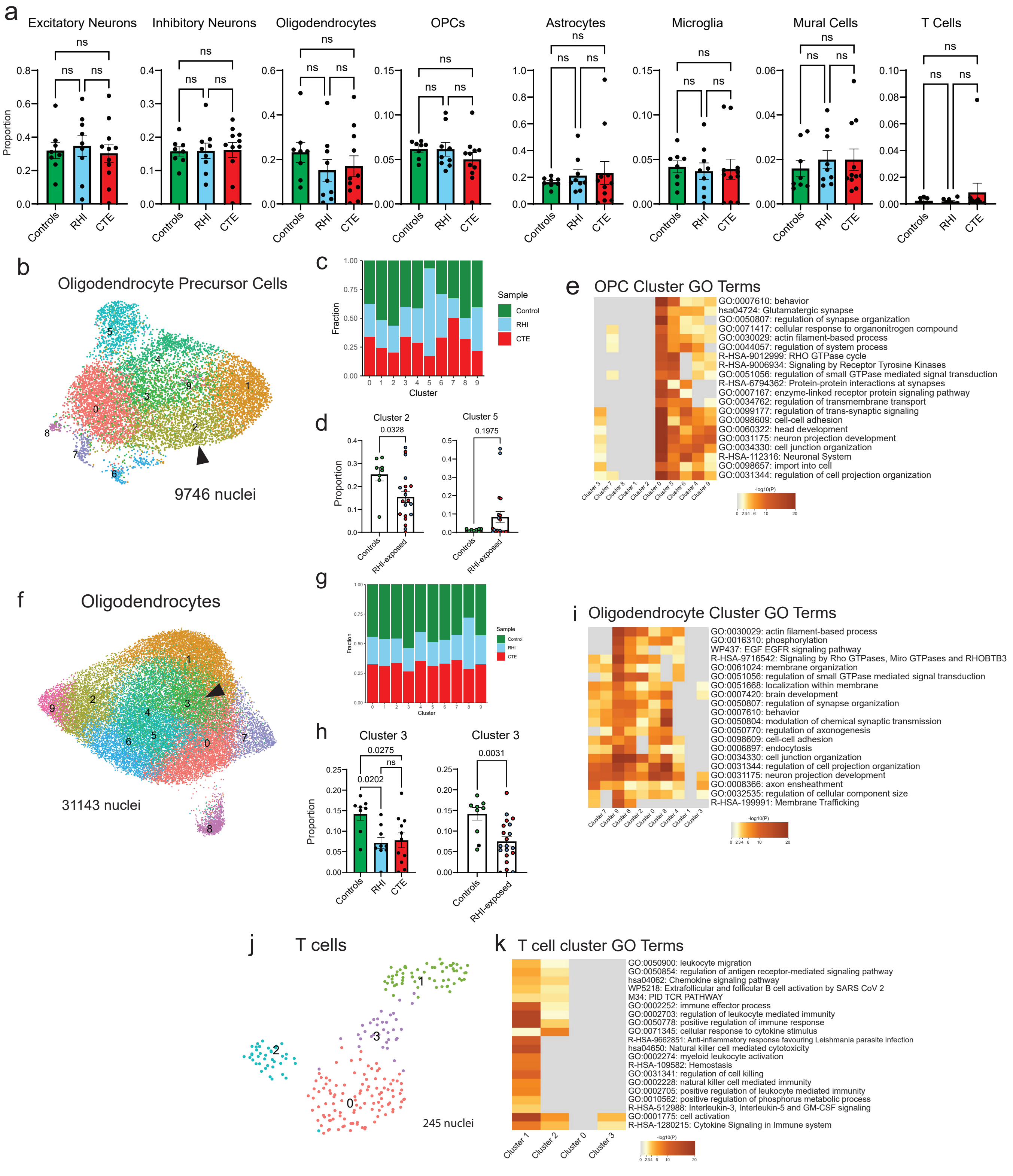

a
Excitatory Neurons
Inhibitory Neurons
Oligodendrocytes
OPCs
Astrocytes
Microglia
Mural Cells
T Cells
ns
Proportion
Controls
RHI
CTE
b
Oligodendrocyte Precursor Cells
9746 nuclei
c
Fraction
Cluster
Sample
Control
RHI
CTE
d
Cluster 2
Cluster 5
0.0328
0.1975
Proportion
Controls
RHI-exposed
e
OPC Cluster GO Terms
GO:0007610: behavior
hsa04724: Glutamatergic synapse
GO:0050807: regulation of synapse organization
GO:0071417: cellular response to organonitrogen compound
GO:0030029: actin filament-based process
GO:0044057: regulation of system process
R-HSA-9012999: RHO GTPase cycle
R-HSA-9006934: Signaling by Receptor Tyrosine Kinases
GO:0051056: regulation of small GTPase mediated signal transduction
R-HSA-6794362: Protein-protein interactions at synapses
GO:0007167: enzyme-linked receptor protein signaling pathway
GO:0034762: regulation of transmembrane transport
GO:0099177: regulation of trans-synaptic signaling
GO:0098609: cell-cell adhesion
GO:0060322: head development
GO:0031175: neuron projection development
GO:0034330: cell junction organization
R-HSA-112316: Neuronal System
GO:0098657: import into cell
GO:0031344: regulation of cell projection organization
Cluster 3
Cluster 7
Cluster 8
Cluster 1
Cluster 2
Cluster 0
Cluster 5
Cluster 6
Cluster 4
Cluster 9
-log10(P)
f
Oligodendrocytes
31143 nuclei
g
Fraction
Cluster
Sample
Control
RHI
CTE
h
Cluster 3
Cluster 3
0.0275
ns
0.0202
0.0031
Proportion
Controls
RHI
CTE
RHI-exposed
i
Oligodendrocyte Cluster GO Terms
GO:0030029: actin filament-based process
GO:0016310: phosphorylation
WP437: EGF EGFR signaling pathway
R-HSA-9716542: Signaling by Rho GTPases, Miro GTPases and RHOBTB3
GO:0061024: membrane organization
GO:0051056: regulation of small GTPase mediated signal transduction
GO:0051668: localization within membrane
GO:0007420: brain development
GO:0050807: regulation of synapse organization
GO:0007610: behavior
GO:0050804: modulation of chemical synaptic transmission
GO:0050770: regulation of axonogenesis
GO:0098609: cell-cell adhesion
GO:0006897: endocytosis
GO:0034330: cell junction organization
GO:0031344: regulation of cell projection organization
GO:0031175: neuron projection development
GO:0008366: axon ensheathment
GO:0032535: regulation of cellular component size
R-HSA-199991: Membrane Trafficking
Cluster 7
Cluster 9
Cluster 6
Cluster 2
Cluster 0
Cluster 8
Cluster 4
Cluster 1
Cluster 3
-log10(P)
j
T cells
245 nuclei
k
T cell cluster GO Terms
GO:0050900: leukocyte migration
GO:0050854: regulation of antigen receptor-mediated signaling pathway
hsa04062: Chemokine signaling pathway
WP5218: Extrafollicular and follicular B cell activation by SARS CoV 2
M34: PID TCR PATHWAY
GO:0002252: immune effector process
GO:0002703: regulation of leukocyte mediated immunity
GO:0050778: positive regulation of immune response
GO:0071345: cellular response to cytokine stimulus
R-HSA-9662851: Anti-inflammatory response favouring Leishmania parasite infection
hsa04650: Natural killer cell mediated cytotoxicity
GO:0002274: myeloid leukocyte activation
R-HSA-109582: Hemostasis
GO:0031341: regulation of cell killing
GO:0002228: natural killer cell mediated immunity
GO:0002705: positive regulation of leukocyte mediated immunity
GO:0010562: positive regulation of phosphorus metabolic process
R-HSA-512988: Interleukin-3, Interleukin-5 and GM-CSF signaling
GO:0001775: cell activation
R-HSA-1280215: Cytokine Signaling in Immune system
Cluster 1
Cluster 2
Cluster 0
Cluster 3
-log10(P)
