# Supplementary material for: Repetitive head impacts induce neuronal loss and neuroinflammation in young athletes
Source: bioRxiv. 2024 Mar 28:2024.03.26.586815. Preprint. [Version 1] doi: 10.1101/2024.03.26.586815 (PMC10996668; doi:10.1101/2024.03.26.586815)
Supplement: Supplementary file 2 [file media-2.pdf]

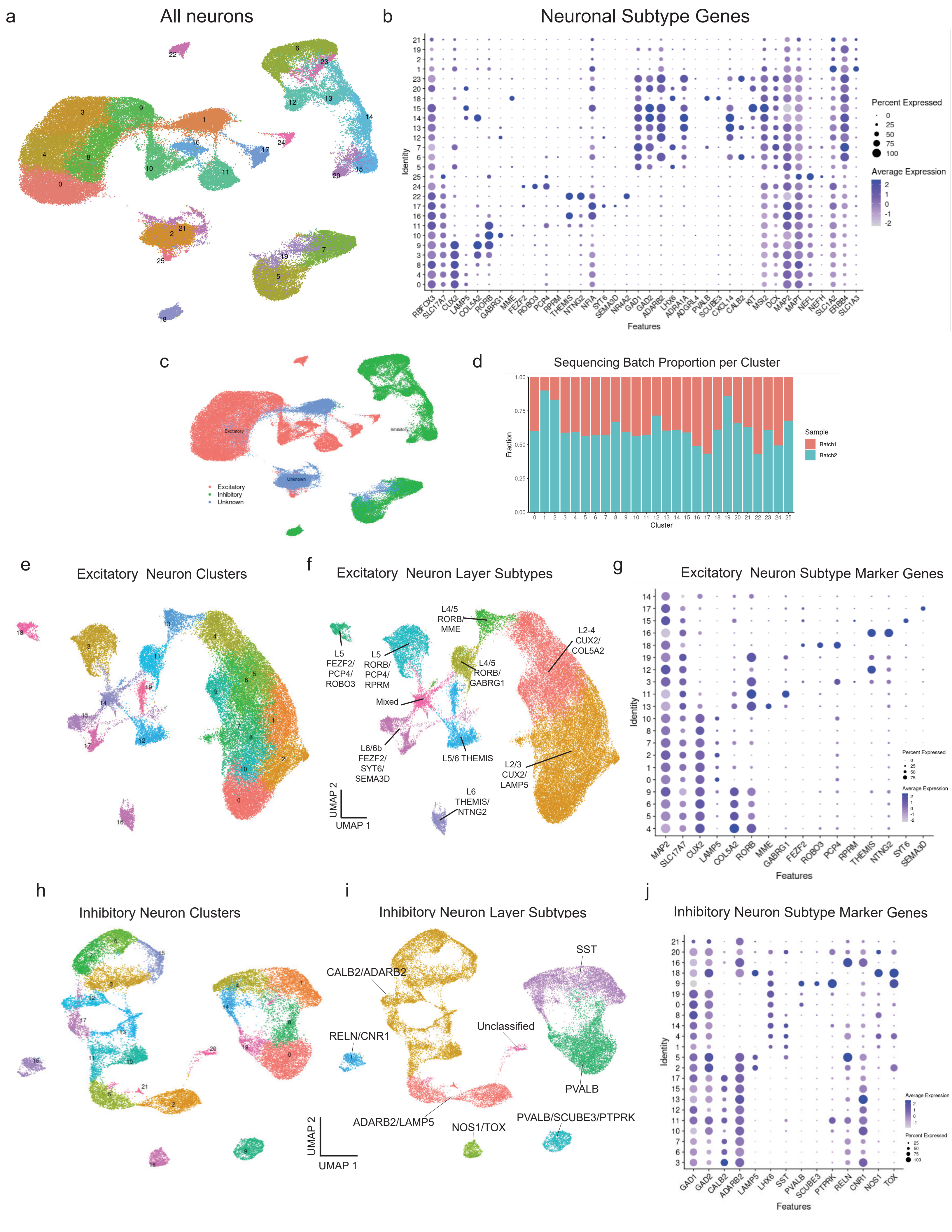
a
All neurons
b
Neuronal Subtype Genes
Percent Expressed
Average Expression
Identity
Features
RBFOX3
SLC17A7
CUX2
LAMP5
COL5A2
RORB
GABRG1
MME
FEZF2
ROBO3
PCP4
RPRM
THEMIS
NTNG2
NFIA
SYT6
SEMA3D
NR4A2
GAD1
GAD2
ADARB2
LHX6
ADRA1A
ADGRL4
PVALB
SCUBE3
CXCL14
CALB2
KIT
MSI2
DCX
MAP2
MAPT
NEFL
NEFH
SLC1A2
ERBB4
SLC1A3
c
Excitatory
Inhibitory
Unknown
d
Sequencing Batch Proportion per Cluster
Fraction
Cluster
Sample
Batch1
Batch2
e
Excitatory Neuron Clusters
f
Excitatory Neuron Layer Subtypes
L4/5 RORB/ MME
L2-4 CUX2/ COL5A2
L5 FEZF2/ PCP4/ ROBO3
L5 RORB/ PCP4/ RPRM
L4/5 RORB/ GABRG1
Mixed
L6/6b FEZF2/ SYT6/ SEMA3D
L5/6 THEMIS
L2/3 CUX2/ LAMP5
L6 THEMIS/ NTNG2
UMAP 2
UMAP 1
g
Excitatory Neuron Subtype Marker Genes
Identity
Features
MAP2
SLC17A7
CUX2
LAMP5
COL5A2
RORB
MME
GABRG1
FEZF2
ROBO3
PCP4
RPRM
THEMIS
NTNG2
SYT6
SEMA3D
Percent Expressed
Average Expression
h
Inhibitory Neuron Clusters
i
Inhibitory Neuron Layer Subtypes
CALB2/ADARB2
SST
Unclassified
RELN/CNR1
PVALB
ADARB2/LAMP5
NOS1/TOX
PVALB/SCUBE3/PTPRK
UMAP 2
UMAP 1
j
Inhibitory Neuron Subtype Marker Genes
Identity
Features
GAD1
GAD2
CALB2
ADARB2
LAMP5
LHX6
SST
PVALB
SCUBE3
PTPRK
RELN
CNR1
NOS1
TOX
Average Expression
Percent Expressed
