# Supplementary material for: Repetitive head impacts induce neuronal loss and neuroinflammation in young athletes
Source: bioRxiv. 2024 Mar 28:2024.03.26.586815. Preprint. [Version 1] doi: 10.1101/2024.03.26.586815 (PMC10996668; doi:10.1101/2024.03.26.586815)

a

HALO AI identification of Nissl+ Neurons

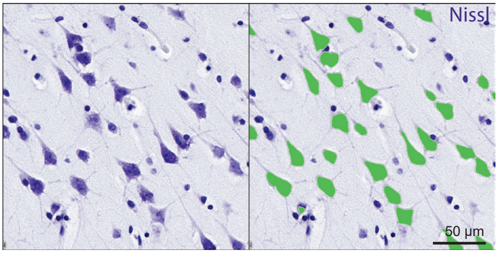

Supplement: Supplementary file 3 [file media-3.pdf]
